# Phytotoxicity and Indole Acetic Production by Rhizobacterial Strains

**DOI:** 10.12688/f1000research.174474.1

**Published:** 2026-01-10

**Authors:** Damilola O. Abiodun, Tolulope A. Ogunnusi, Oghenerobor B. Akpor

**Affiliations:** 1 Biological Sciences, Afe Babalola University, Ado Ekiti, Ekiti, Nigeria

**Keywords:** Deleterious rhizobacteria, culture age, indole acetic acid, phytotoxicity

## Abstract

**Background:**

Deleterious rhizobacteria (DRB) constitute an emerging category of bioherbicides with the capacity to inhibit weed germination through phytotoxic metabolites.

**Aim:**

This study aimed to isolate and screen rhizobacteria for deleterious potentials. Bacterial isolation was carried out using the standard pour plating method.

**Methods:**

Five agricultural crops (cucumber, soybean, sorghum, cowpea and maize) were assayed for phytotoxic screening against the DRB. In addition, antimicrobial potential of the DRB against test pathogens and indole acetic acid (IAA) were also investigated. Polymerase chain reaction and gene sequencing of the isolates revealed three strains of *Pseudomonas aeruginosa* and one each of *P. fluorescens* and *E. hormaechei.*

**Results:**

The antimicrobial outcomes of the test DRB revealed broad-spectrum inhibition against the test pathogens. In terms of culture age, younger cultures (≤48 h) mostly stimulated or matched control germination and vigor indices with toxicity increasing progressively with age beyond 120 h for most of the seeds. In the case of steeping duration, short steeping durations (1–2 h) typically maintained germination and vigor near control levels. Progressive extension of steeping time increased inhibition across all isolates. At different inoculum concentrations, higher inoculum concentrations produced stronger inhibitory effects than diluted suspensions. Full-strength inoculum (100%) suppressed vigor drastically, whereas 20–40% dilutions produced diminished or reversible effects. Also, all isolates produced measurable IAA in tryptophan-supplemented media with output increasing with incubation age, correlating with suppression of vigor at later time points.

**Conclusion:**

The indigenous origin of the DRB evaluated in this study enhances environmental compatibility and reduces ecological disturbance risks.

## 1. Introduction

Globally, food security and economic growth are dependent on agricultural activities. The growing demand for food and agricultural products and the necessity to enhance agricultural productivity have led to the adoption of agrochemicals, such as chemical fertilizers, pesticides and herbicides ( Hussain *et al., *2015).

Weeds are plant pest that affect the productivity leading to loss of plant yield ( Hossain *et al., *2020). They are unwanted plants, which cause severe limitations in crop production. Their presence can cause harmful effects on plants as they can serve as hosts of pests and diseases causing competition for light, water carbon dioxide, space and other essential growth nutrients with the main crop ( Beche *et al., *2023; Paul *et al., *2024).

Current agricultural management practices tend towards the use of agrochemicals. Herbicides are very important in the control of weeds which are one of the most destructive and persistent biotic limitations to crop production Synthetic chemical herbicides were introduced in the middle of the 20th century as an agricultural management practice providing fast and effective methods of managing the unwanted plants. Glyphosate, paraquat and atrazine are common herbicides that are used in modern agriculture. Chemical herbicides are effective, but continuous and indiscriminate use of these chemicals cause long-term consequences of environmental contamination in soil and water, resistance of weed and its residues in agricultural products poses harm to consumer health and beneficial microorganisms and aquatic life ( Chtourou *et al., *2024). Besides, long-term exposures to herbicidal residues have been linked to human health risks, including endocrine disruption, carcinogenicity and neurological disorders ( Mhlongo *et al., *2022).

Owing to the disadvantages and environmental consequences of chemical herbicide, it is therefore important to develop a more effective, environmentally friendly and sustainable agricultural management practice that minimizes the dependence on the use of chemical herbicides while maintaining high productivity. A promising biological control approach to sustainable agriculture is the use of deleterious rhizobacteria (DRB). They are a group of soil bacteria that can suppress the growth of plant by colonizing the root surface of the seedlings and producing phytotoxins that inhibit development by reducing plant density, biomass and seedling germination. They are an example of biological, eco-friendly and sustainable agricultural control. They are influenced by harmful rhizobacteria by the overproduction of phytotoxins like hydrogen cyanide (HCN) and indole acetic acid (IAA), which they can absorb, competition for resources and space and disruption of hormone signaling. Deleterious rhizobacteria have the potential to target specific plant species without having a detrimental effect on non-target crops or other beneficial microorganisms, which makes them beneficial biocontrol agents ( Hoesain *et al., *2024). *Pseudomonas aeruginosa*, *Pseudomonas fluorescens, Erwinia herbicola, Alcaligenes spp.* and *Pseudomonas syringae* are some rhizobacteria that have been described as bioherbicides. Deleterious rhizobacteria provide an eco-friendly alternative to synthetic herbicides because they are able to adapt to changes in the environment, remain in the soil without accumulating toxic metabolites and coexist with beneficial soil organisms ( Mehmood *et al., *2023).

The problem of weed control in agricultural practice poses a major global challenge to agricultural sustainability and improving crop yield ( Ramesh *et al., *2017). The increase in the global population over the last several decades has imposed pressure on agricultural land to increase crop yield simultaneously by controlling the growth of wanted plants. This has contributed to the overuse of chemical herbicides. Although synthetic herbicides are effectively used in modern agriculture, the problem with their use is that they lead to increase the level of accumulation of heavy metals, destruction of biodiversity in the soil, production of herbicide-resistant types of weeds, environmental degradation, and potential health hazards ( Kostina-Bednarz *et al., *2023). The consistent use of these chemical herbicides can make the soil fragile, losing the soil structure, decreasing soil aeration and activities of the soil macrofauna ( Frimpong *et al., *2018). Heap, (2014) reported that weeds have evolved resistance to twenty-one (21) out of the twenty-five (25) known herbicide sites of action and to 152 different herbicides. Some weeds have developed multiple herbicide resistance. Due to these challenges, it is important to explore other sustainable and eco-friendly approaches to control weeds that minimizes the dependence on chemical herbicide use. The application of deleterious rhizobacteria appears to be a sustainable biological method of weed management through various mechanisms which could provide promising and eco-friendly approach. In addition, there is paucity of information on the diversity, specificity, and weed-suppressive mechanisms of DRB, and their practical application as bioherbicides. Moreover, there is lack of systematic screening and characterization of DRB strains from different agroecological environments limits the development of effective, targeted bioherbicidal formulations. Therefore, this study seeks to address this knowledge gap by isolating, screening and evaluating different rhizobacteria species isolated from various soil for DRB potential. This study aimed to isolate and screen rhizobacteria for deleterious potentials and assess their herbicidal efficacy.

## 2. Materials and methods

### 2.1 Isolation of rhizobacteria

The rhizobacterial strains used in this study were isolated from rhizosphere soils within Afe Babalola University Teaching and Research Farm, Ado Ekiti, Ekiti State, Nigeria.

For isolation, 100 g of soil (5 cm depth) were collected with sterile metal spatula into sterile polythene bags and carefully transported to the laboratory for serial dilution. Serial dilution was carried out by dissolving 15 g of sterile in 150 mL of normal saline (0.85 NaCl w/v) solution, followed by 10-fold serial dilution in sterile distilled water.

Isolation of bacterial was done following the standard pour plate method. 1 mL of a known dilution was dispensed in sterile Petri dish, 15 mL of the pre-cooled sterilised nutrient agar was dispensed and properly mixed. The Petri dishes were allowed to dry before incubating at 35°C±2 °C for 24 h. Following incubation, representative colonies were subcultured on nutrient agar plates to obtain pure cultures, which were kept on nutrient agar slants at 4°C±2 °C till when required.

### 2.2 Test seeds

Seeds of five (5) crops comprising of cucumber ( *Cucumis sativus*), soybean ( *Glycine max*), sorghum ( *Sorghum bicolor*), cowpea ( *Vigna unguiculata*) and maize ( *Zea mays*) obtained from a local market in Ado Ekiti, Ekiti State, Nigeria were used for this study.

The test seeds were identified and authenticated by Mr Omotayo (Taxonomist) at the Ekiti State University Herbarium, Ado Ekiti, Ekiti State, Nigeria. Samples of the respective seeds were deposited at the Herbarium and voucher numbers obtained accordingly: UHAE 2025057, UHAE 2025058, UHAE 2025059, UHAE 2025060 and UHAE 2025061 for the cucumber, soybean, sorghum, cowpea and maize, respectively.

### 2.3 Preliminary phytotoxicity and antimicrobial screening

Prior to use, test seeds used were surface-sterilized with sodium hypochlorite (5% v/v) solution for 5 min and rinsed three times in distilled water. All seed lots used for this study were tested for viability according as described by Akpor *et al., *(2022). For viability testing, approximately 200 surface-sterilized seeds from a lot were placed in a 500 mL-distilled water beaker and observed for floated seeds. The seeds that floated were regarded as non-viable and discarded. Preliminary screening for phytotoxic effect for the bacteria isolates was carried out following the method described by Akpor *et al., *(2022). The respective seeds were soaked in 24-h-old nutrient broth cultures of the respective test isolates and allowed to stand for 2 h. Seven (7) seeds were withdrawn and planted in Petri dishes containing 3.48 g of cotton wool.

The setups were incubated under fluorescent light for 7 d ambient temperature monitored daily for growth germination. At the end of the 7 d incubation period, shoot lengths of the respective seeds were measured and vigor index calculated accordingly:

Vigor index=Average final germinationXAverage shoot.

Antimicrobial screening was carried out using 24 h old cultures of the test bacterial isolates against selected pathogens ( *Salmonella typhi, Bacillus subtilis, Bacillus proteolyticus, Xanthomonas campestris, Pseudomonas aeruginosa, Klebsiella pneumonia* and *Pseudomonas fuscoginae*) using the agar well diffusion method ( Akpor *et al., *2022).

To a 200 mL of sterile molten nutrient agar was inoculated 1 mL of 24 h old cultures of a respective test pathogen and thoroughly mixed. After mixing, 20 mL of agar containing the respective pathogens were dispensed into plates and allowed to dry. Upon solidifying, a sterile cork borer was used to bore four holes into the agar plates containing the test pathogen. Using a sterile micropipette, approximately 100 uL of the 24 h old broth cultures of a test rhizobacterial inoculum was added to a bored hole and left to diffuse. The inoculated plates were then incubated at 37°C for 24 h to observe for clear zones. The antimicrobial potential of an isolate against a test pathogen was ascertained by observing for zones of inhibition Zones of inhibition were measured using a transparent metre rule in millimetres.

Only bacterial inoculums that revealed reduced vigor index of at least three of the test plants and inhibited at least three of the test pathogens screened against were selected for further studies.

### 2.4 Characterization of isolates

Characterization of rhizobacterial strains was carried out using 16 S rRNA partial gene sequencing and polymerase chain reaction (PCR) techniques. DNA extraction was carried out using 48 h-old broth cultures grown from single colonies on nutrient agar ( Wang *et al., *2011). The resultant cultures were centrifuged for 5 min at 4600 g to extract the pellet. Pellets obtained were suspended in 520 μl of Tris-EDTA buffer (10 mM trisHcl, 1 mM EDTA, pH 8.0), 15 μl of 20% Sodium Dodecyl Sulfate and 3 μl of Proteinase K (20 mg/ml) were added. This sample was incubated at 37°C for 1 h and then 100 μl of 5 M sodium chloride and 80 μl of 10% cetyltrimethylammonium bromide solution in 0.7M sodium chloride were added and vortexed. Also, the mixture was incubated at 65°C for 15 min and 10 min on ice which was then centrifuged at 7200 g for 20 min after an addition of chloroform: isoamyl alcohol (24:1) and precipitation of DNA was done after the addition of isopropanol (1:0.6) at -20 °C for 16 h. The mixture was centrifuged at 13000 g for 10 min, resultant DNA was washed with 500 μl of 70% ethanol, left to dry under room temperature for 3 h and then dissolved in 50 μl of Tris-EDTA buffer.

The cocktail of PCR sequencing comprised of 10 μl 5x GoTaq colourless reaction, 3 μl 25mM MgCl2, 1 μl 10 mM mixture of dNTPs, 1 μl 10 pmol each of 27F 5′- AGA GTT TGA TCM TGG CTC AG-3′ and - 1525R, 5′-AAGGAGGTGATCCAGCC-3′ primers and 0.3 units of Taq DNA polymerase (Promega, USA) made up to 42 μl with sterile distilled water 8 μl DNA template.

The PCR was carried out in a GeneAmp 9700 PCR System Thermalcycler (Applied Biosystem Inc., USA) with a PCR profile that consisted of an initial denaturation at 94°C for 5 min, followed by 30 cycles consisting of 94°C for 30 s, 50°C for 60 s and 72°C for 90 s and a final termination at 72°C for 10 min ( Innis *et al., *2012).

Agarose gel of 1% was ran to check the integrity of the amplified gene fragment to confirm amplification. The base pairs were separated on a 1XTAE buffer that was already prepared and this was used to prepare 1.5% agarose gel before boiling in the microwave for 5 min. This molten agarose was then cooled to 60°C and 3 μl of 0.5 g/ml ethidium bromide added, followed by insertion of a comb into the slots of the casting tray and the pouring of the molten agarose into the tray. The gel was then left to dry over a period of 20 min to create the wells and 1XTAE gel loading buffer was poured into the gel tank to barely submerge the gel. 100 bp DNA ladder was contained into well 1 after which 2 μl of 10X blue gel loading dye was loaded into the gel. The gel was subjected to electrophoresis for 45 min at 120 V and examined with ultra-violet trans-illumination and photographed. The sizes of the PCR products were estimated through their comparison with the mobility of 100 bp molecular weight ladder which was also run with experimental samples in the gel.

Following integrity of the gel, to eliminate the reagents of the PCR, purification was carried out with ethanol on the amplified fragments. 7.6 μl of 3 M Na acetate and 240 μl of 95% ethanol were added to about 40 μl of PCR amplified product in a sterile eppendorf tube and mixed thoroughly by vortexing before incubation at -20 °C for 30 min. The mixture was further centrifuged at 13000 g and 4°C for 10 min. This was succeeded by the removal of the cell-free supernatant after which the pellet was washed by the addition of 150 μl of 70% ethanol and later centrifuged at 7500 g and 4°C for 15 min.

After centrifuging, resulting cell-free supernatant was discarded by inverting the tubes into a waste container and the tubes were then subsequently inverted on a cotton paper to dry in a fume cupboard for 10 to 15 min at ambient temperature. The pellet was again suspended in 20 μl of sterile distilled water and kept at −20°C prior to sequencing. In order to verify the purified product, the purified fragment was resolved on a 1.5% agarose gel that was run at 110 V in 1 h before quantifying with nanodrop (model 2000 of thermo scientific).

Sequencing of the amplified fragments was performed using Genetic Analyzer 3130xl sequencer from Applied Biosystems using manufacturers’ manual and with a BigDye terminator v3.1 cycle sequencing kit. All genetic analysis was done using Bio- Edit software and MEGA 6. The sequences were submitted to the National Centre of Biotechnology Information (NCBI) database and accession numbers acquired.

### 2.5 Phytotoxicity studies

To determine the effect of inoculum age, pure culture of respective rhizobacterial strains was inoculated into seven 150 mL-capacity conical flasks, containing 100 mL sterile nutrient broth and incubated at 35°C ± 2°C. Every 24 h, for a 7-d duration, a flask containing the inoculum was withdrawn and the inoculum used for steeping of the seeds for 2 h before planting in Petri dishes containing blotters and incubated, as described in section 3.3.

Following planting, seeds were monitored daily for % germination for a 7 d duration. When the incubation period was over, germination rate, germination time and vigor index were calculated: ( Leggatt *et al., *1949) ( Ellis *et al., *1986).

$$\%\text{germination}=\frac{\text{Total}\;\mathrm{no}\;\text{of germinated seeds}}{\text{Total}\;\mathrm{no}\;\text{of sown seeds}2!}X\;100\%.$$

$$\text{Mean germination time}\;\left(\mathrm{MGT}\right)=\Sigma \frac{\mathrm{fx}}{f}.$$

Where f is the number of seeds germinated on day x ( Salehzade *et al., *2009).

Germination index(GIX)=(7×N1)+(6×N2)+(5×N3)+……+(1×N7)

Where N1, N2, N3…N7 stand for the number of seeds that sprouted on the first, second, and third days up until the seventh day ( Abdul-Baki & Anderson, 1973).

Vigor index=Final%germinationXAverage plant height.

To determine the effect of steeping time length, known culture age of a respective test bacterial strain was used for steeping. Each of the test seeds was soaked in an inoculum and taken out every 1 h for a period of 5 h for planting and incubated as described earlier. Following planting, % germination, germination time, germination rate and vigor index were calculated as earlier described.

With regard to the effect of inoculum concentration, test seeds were soaked in varying concentrations of a respective inoculums of known culture age and steeped for 2 h before planting and incubation for a 7-d duration. The inoculum dilutions used for steeping were 100:0, 80:20, 60:40, 40:60, 20:80 and 100:0, inoculum to water ratios, respectively. Following planting, % germination, germination rate, germination time and vigor index were calculated as described earlier.

### 2.6 Indole acetic acid production

Production of indole acetic acid (IAA) by the test isolates was determined using a medium supplemented with and without of tryptophan, respectively.

Indole acetic acid (IAA) was assessed using the colorimetric procedure, following the addition of Salkowski reagent as described by Sarker and Al-Rashid (2013). For the preparation of Salkowski reagent, 1.85 g of 0.5 M ferric chloride (FeCl _3_) was mixed with 40 mL of 70% perchloric acid (HClO _4_).

The solution was thoroughly mixed and kept in a dark container to avoid photodegradation. After incubation at respective inoculum ages (24 h, 48 h, 72 h, 96 h, 120 h, 144 h and 168 h), bacterial culture was centrifuged at 4000 rpm for 10 min to separate bacterial cells and the supernatant. Without leaving any cell debris behind, the supernatant was carefully decanted in a sterile bottle. To perform colorimetric assay, 2 mL of freshly prepared Salkowski reagent was added to 10 mL of the supernatant, mixed gently and left to react for 5 mins without direct light to maximize colour development. Readings were recorded using a spectrophotometer at wavelength of 530 nm. A blanking procedure was done with a control sample consisting of the uninoculated medium only and Salkowski reagent prior to the reading and recording of absorbance values of all the test samples read and recorded. Known concentrations of IAA standards in the same medium and reagent conditions were used to prepare calibration curve. To prepare a calibration curve, a plot of the absorbance against IAA concentrations was made. The absorbance values of samples were then extrapolated using the calibration curve to determine the IAA concentration in them. For the calibration curve, R ^2^ value of 0.9481 was obtained.

### 2.7 Extraction and characterization of metabolites

Crude metabolites of the isolates were extracted using the cold extraction method as reported by Akpor *et al., *(2022). For extraction of the cell free supernatant (CFS), a 168 h-old broth cultured in 200 mL of nutrient broth of a test bacterial strain was centrifuged for 15 min at 5000 rpm. The acidic solution was kept overnight in a refrigerator at 4°C. Ethyl acetate and methanol (3:1 v/v) solvent mixture was added to extract the precipitated metabolite. The crude metabolites were obtained by decantation and evaporated by placing the beaker in a water bath after which it was dried and the contents quantified then stored in sterile universal bottles in a refrigerator until when needed.

Gas Chromatography Mass Spectroscopy (GC-MS) and Fourier Transformed Infrared (FT-IR) Spectroscopy methods were used to characterize the extracted metabolites.

A Varian 3800/4000 gas chromatograph mass spectrometer with a non-polar fused silica capillary column (30 m x 0.25 mm, 0.25 μm film thickness) was used to perform GC-MS analysis. The temperature of the oven was set at 70°C for 4 min and then set to a final temp of 240°C at a rate of 8°C/min and was maintained at that temperature at 20 min. Flow rate of Nitrogen carrier gas was 1mL/min. The volume of the injection was 1 μL of hexane-diluted oil. Electron ionization mode was employed in the mass spectrometer detector and all the spectra obtained with settings of mass range of 40 to 800 m/z and automatic gain control. The compounds were identified using the match of the retention time and the mass spectra against the standards and the NIST mass spectra library.

Computer searches of the National Institute Standard and Technology (NIST) Version 08 data library were used to identify compounds contained in a metabolite. The GC-MS interpretation on mass spectrum was performed by the database of NIST which contains over 62,000 patterns. The mass spectra of the unknown component and the known components stored at NIST library were compared. Components of the test materials were identified in terms of name, molecular weight and structure.

For FTIR analysis, the extracted metabolite was combined with potassium bromide in a mixing ratio of 1:100. To obtain a 13 mm pellet, the combined powder was compressed in a dye at a load of 10 tons. The pellet was subsequently placed in the FTIR chamber to be analyzed and washed with 10 mL of demineralized water to remove free proteins/enzymes. Following drying and grinding with potassium bromide (KBr) pellets, the samples were then analyzed. The transmittance axis of the graph was plotted using the reference material of potassium bromide. In the FITR spectroscopy, the transmittance versus wavelength provided the data.

### 2.8 Statistical analysis

The Statistical Software of Social Scientists (SPSS) version 26.0 computer software was used to perform all statistical analysis. All data were expressed as standard deviation and means of triplicate analysis. One-Way Analysis of Variance (ANOVA) test was used to compare means and the Least Significant Difference (LSD) test was used to compare the means of multiple comparisons. All the analyses were conducted at 0.05 probability level.

## 3. Results

### 3.1 Rhizobacterial strains

A total of 43 rhizobacterial strains were isolated from rhizospheres within Afe Babalola University, Nigeria. The isolates were screened preliminarily for antimicrobial potentials against selected pathogens and phytotoxicity potentials. Isolates that showed antimicrobial activity against at least three of the test pathogens and phytotoxic activity (evidenced through reduced vigor index) against a minimum of three selected seeds (cowpea ( *Vigna unguiculata*), soybean ( *Glycine max*), maize ( *Zea mays*), cucumber ( *Cucumis sativus*) and sorghum ( *Sorghum bicolor*)) were used for further studies. A total of five rhizobacterial strains, which consists of three strains of *Pseudomonas aeruginosa,* one strain of *Enterobacter hormaechei* and one strain of *Pseudomonas fluorescens* were used for further studies ( Table 1).

**Table 1.** National Centre for Biotechnology Information (NCBI) Blast showing the identity of the sequenced organism.

Sample ID	Scientific name	Max score	Total score	Query cover	E value	Percent identity	Accession
D1	*Pseudomonas aeruginosa*	2161	2161	100%	0	99.49%	PV936735
D2	*Pseudomonas aeruginosa*	2189	2189	100%	0	99.92%	PV936736
D3	*Pseudomonas fluorescens*	2170	2170	99%	0	99.58%	PV936737
D4	*Enterobacter hormaechei*	2194	2194	100%	0	99.83%	PV936738
D5	*Pseudomonas aeruginosa*	2200	2200	100%	0	100.00%	PV936739

### 3.2 Antibacterial activity

Antibacterial potentials of the rhizobacterial inoculums revealed variation in the degree of inhibition against the test pathogens. Overall, the highest zones of inhibition were observed against *P. aeruginosa* and *P. fuscoginae* (27.5 mm) in the presence of *P. aeruginosa* (PV936735). In the case of *P. aeruginosa* (PV936736), *P. fluorescens* (PV936737) and *E. hormaechei* (PV936738), highest zones of inhibition of 26.5 mm and 25.0 mm and 31.5 mm were recorded against *Bacillus subtilis,* respectively. For *P. aeruginosa* (PV936739), the highest value was recorded against *K. pneumoniae,* with zone of inhibition of 28.0 mm ( Table 2).

**Table 2.** Antibacterial potentials of the rhizobacterial inoculums against selected bacterial pathogens.

Rhizobacterial inoculums	Test pathogens	Mean zones of inhibition
*P. aeruginosa* (PV936735)	*Salmonella typhi*	-
	*Bacillus subtilis*	-
	*Bacillus proteolyticus*	22.5 ± 3.54
	*Xanthomonas campestris*	23.5 ± 2.12
	*Pseudomonas aeruginosa*	27.5 ± 3.54
	*Klebsiella pneumoniae*	17.5 ± 3.54
	*Pseudomonas fuscoginae*	27.5 ± 3.54
*P. aeruginosa* (PV936736)	*Salmonella typhi*	23.0 ± 2.83
	*Bacillus subtilis*	26.5 ± 2.12
	*Bacillus proteolyticus*	-
	*Xanthomonas campestris*	20.5 ± 0.71
	*Pseudomonas aeruginosa*	15.0 ± 0
	*Klebsiella pneumoniae*	-
	*Pseudomonas fuscoginae*	-
*P. fluorescens* (PV936737)	*Salmonella typhi*	-
	*Bacillus subtilis*	25.0 ± 0
	*Bacillus proteolyticus*	-
	*Xanthomonas campestris*	-
	*Pseudomonas aeruginosa*	22.5 ± 3.54
	*Klebsiella pneumoniae*	20.0 ± 7.07
	*Pseudomonas fuscoginae*	-
*E. hormachei* (PV936738)	*Salmonella typhi*	19.0 ± 8.49
	*Bacillus subtilis*	31.5 ± 4.95
	*Bacillus proteolyticus*	-
	*Xanthomonas campestris*	-
	*Pseudomonas aeruginosa*	-
	*Klebsiella pneumoniae*	27.5 ± 3.54
	*Pseudomonas fuscoginae*	-
*P. aeruginosa* (PV936739)	*Salmonella typhi*	-
	*Bacillus subtilis*	22.5 ± 3.54
	*Bacillus proteolyticus*	-
	*Xanthomonas campestris*	21.0 ± 1.41
	*Pseudomonas aeruginosa*	-
	*Klebsiella pneumoniae*	28.0 ± 0
	*Pseudomonas fuscoginae*	-

Values are mean of duplicate samples. ‘-’ represents no observed inhibition.

### 3.3 Phytotoxicity assay

**3.3.1 Effect of culture age**

***Cowpea***

Overall, the data showed that young bacterial cultures enhanced rapid germination and high seedling vigor, whereas cultures aged beyond 120 h increasingly delayed germination and suppressed vigor, with the severity of phytotoxicity varying across strains. Among the isolates, *P. aeruginosa* PV936735 showed high germination up to 120 h but the vigor reduced sharply at 144 h, signifying phytotoxic activity. *P. aeruginosa* PV936736 preserved germination near 100% while vigor decreased steadily after 120 h. In the presence of *P. fluorescens*, excellent early vigor was displayed with younger culture while older cultures showed moderate, remaining above the control. For *E. hormaechei*, consistently high germination and vigor was maintained across all culture ages, indicating the least phytotoxic effect ( Table 3).

**Table 3.** Effect of culture age on phytotoxicity of the cowpea seeds in presence of the test bacterial strains.

Incubation time	% germination	Germination rate	Germination time	Vigor index
***Pseudomonas aeruginosa* (PV936735)**				
24 h	85.71 ± 14.29 ^b^	0.24 ± 0 ^a^	4.14 ± 0.05 ^a^	1439.05 ± 548.31 ^b^
48 h	100 ± 0 ^a^	0.23 ± 0.01 ^b^	4.34 ± 0.09 ^a^	1717.62 ± 341.84 ^b^
72h	95.24 ± 8.25 ^a^ ^b^	0.24 ± 0 ^a^	4.41 ± 0.29 ^a^	614.01 ± 318.36 ^c^
96 h	100 ± 0 ^a^	0.21 ± 0 ^c^	4.76 ± 0.05 ^b^	1380 ± 386.95 ^b^
120 h	100 ± 0 ^a^	0.21 ± 0 ^c^	4.83 ± 0.05 ^b^	1430 ± 131.78 ^b^
144 h	95.24 ± 8.25 ^a^ ^b^	0.21 ± 0 ^c^	4.75 ± 0.15 ^b^	587.76 ± 95.54 ^c^
168 h	85.71 ± 0 ^b^	0.21 ± 0 ^c^	4.73 ± 0 ^b^	357.55 ± 32.47 ^c^
***Pseudomonas aeruginosa* (PV936736)**				
24 h	95.24 ± 8.25 ^a^	0.24 ± 0.00 ^a^	4.13 ± 0.11 ^a^	191.99 ± 212.22 ^c^
48 h	100 ± 0 ^a^	0.22 ± 0.00 ^b^	4.53 ± 0.04 ^a^ ^b^	1744.76 ± 188.46 ^a^
72h	100 ± 0 ^a^	0.22 ± 0.00 ^b^	4.46 ± 0.06 ^a^ ^b^	1309.52 ± 174.21 ^b^
96 h	95.24 ± 8.25 ^a^	0.21 ± 0.02 ^b^	4.84 ± 0.48 ^b^	1262.72 ± 282.01 ^b^
120 h	95.24 ± 8.25 ^a^	0.21 ± 0.00 ^b^	4.86 ± 0.09 ^b^	1152.79 ± 130.76 ^b^
144 h	95.24 ± 8.25 ^a^	0.21 ± 0.01 ^b^	4.88 ± 0.20 ^b^	573.33 ± 251.21 ^c^
168 h	100 ± 0 ^a^	0.22 ± 0.00 ^b^	4.60 ± 0.31 ^b^	701.43 ± 272.17 ^c^
***Pseudomonas fluorescens* (PV936737)**				
24 h	100 ± 0 ^a^	0.24 ± 0.01 ^a^	4.22 ± 0.27 ^a^	1872.38 ± 502.23 ^a^
48 h	90.48 ± 8.25 ^b^	0.24 ± 0.01 ^a^	4.23 ± 0.14 ^a^	1657.01 ± 274.91 ^a^
72h	95.24 ± 8.25 ^a^ ^b^	0.24 ± 0.00 ^a^	4.25 ± 0.80 ^a^	1376.80 ± 360.80 ^a^
96 h	100 ± 0 ^a^	0.22 ± 0.00 ^b^	4.63 ± 0.03 ^b^	1541.43 ± 335.04 ^a^
120 h	100 ± 0 ^a^	0.21 ± 0.01 ^b^	4.73 ± 0.19 ^b^	1296.67 ± 421.66 ^a^
144 h	100 ± 0 ^a^	0.22 ± 0.00 ^b^	4.65 ± 0.09 ^b^	773.33 ± 171.22 ^b^
168 h	100 ± 0 ^a^	0.22 ± 0.00 ^b^	4.46 ± 0.09 ^a^	1058.10 ± 348.89 ^b^
***Enterobacter hormaechei* (PV936738)**				
24 h	95.24 ± 8.25 ^a^	0.23 ± 0.01 ^a^	4.26 ± 0.20 ^c^	1692.24 ± 609.15 ^a^
48 h	95.24 ± 8.25 ^a^	0.23 ± 0.01 ^a^	4.31 ± 0.11 ^c^	1526.19 ± 322.14 ^a^
72h	100 ± 0 ^a^	0.23 ± 0.01 ^a^	4.34 ± 0.18 ^c^	1781.43 ± 292.59 ^a^
96 h	100 ± 0 ^a^	0.21 ± 0.00 ^b^	4.71 ± 0.01 ^a^	1572.86 ± 252.39 ^a^
120 h	100 ± 0 ^a^	0.21 ± 0.00 ^b^	4.70 ± 0.15 ^b^	1541.43 ± 108.53 ^a^
144 h	100 ± 0 ^a^	0.20 ± 0.00 ^c^	4.94 ± 0.11 ^a^	824.29 ± 278.62 ^b^
168 h	90.48 ± 8.25 ^b^	0.22 ± 0.01 ^b^	4.56 ± 0.11 ^b^	808.91 ± 170.07 ^b^
***Pseudomonas aeruginosa* (PV936739)**				
24 h	100 ± 0 ^a^	0.25 ± 0.00 ^a^	4.07 ± 0.07 ^a^	1859.05 ± 181.88 ^a^
48 h	80.95 ± 21.82 ^b^	0.23 ± 0.01 ^b^	4.33 ± 0.19 ^b^	854.22 ± 396.39 ^c^
72h	100 ± 0 ^a^	0.23 ± 0.00 ^b^	4.40 ± 0.08 ^b^	1583.81 ± 85.05 ^a^
96 h	100 ± 0 ^a^	0.20 ± 0.01 ^c^	4.98 ± 0.19 ^c^	1458.57 ± 87.00 ^a^
120 h	100 ± 0 ^a^	0.20 ± 0.01 ^c^	5.00 ± 0.12 ^c^	1540.48 ± 51.59 ^a^
144 h	100 ± 0 ^a^	0.22 ± 0.00 ^b^	4.64 ± 0.03 ^b^	880.48 ± 148.78 ^b^ ^c^
168 h	100 ± 0 ^a^	0.22 ± 0.00 ^b^	4.49 ± 0.08 ^b^	1013.33 ± 71.72 ^b^
**Control**	97.28 ± 7.31	0.23 ± 0.01	4.33 ± 0.20	1431.90 ± 475.68

Within columns, values with different and same superscript letters represent differences that are significantly and not significantly different, respectively (p < 0.05).

In the case of germination, most treatments showed high germination percentages (>90%) throughout the culture ages. Treatment with *P. fluorescens* PV936737 consistently matched the control, while *P. aeruginosa* PV936739 sustained full germination after an early decline at 48 h. Significant declines in germination observed in older cultures were evident for *P. aeruginosa* PV936735 (85.7% at 168 h) and for *E. hormaechei* (90.5% at 168 h), suggesting slight inhibition as cultures aged. Germination rate values clustered closely around 0.20–0.24, similar to the control (0.23), reflecting only modest influence of culture age. However, slightly higher rates (0.24–0.25) were noticed with very young cultures (24 h) for most strains, except *E. hormaechei,* indicating a small early-stage stimulation ( Table 3).

With respect to germination time, the control mean germination time was 4.33 d while early cultures (24–48 h) of all strains shortened germination time between 4.1–4.3 d. In the presence of older cultures (96–120 h), extended germination time of between 4.7–5.0 d were recorded, which is an indication of increased phytotoxic activity as cultures aged. The longest germination times were recorded for *P. aeruginosa* PV936739 at 96–120 h.

Vigor index of the cowpea seeds showed peak values (≥1700) with young cultures of *P. fluorescens* (1872 at 24 h), *E. hormaechei* (1781 at 72 h) and *P. aeruginosa* PV936739 (1859 at 24 h). Evidently, most strains showed vertical decline after 120 h, with *P. aeruginosa* PV936735 dipping to 358 by 168 h and *P. aeruginosa* PV936736 to 573, which was well below the control value at 1431.9 ( Table 3).

***Maize***

Among the strains, *P. aeruginosa* PV936735 showed marked drop in vigor and germination after 120 h, suggesting strong phytotoxic metabolites accumulate with culture age while *P. aeruginosa* (PV936736) maintained high germination to 120 h but vigor index fell sharply to 227 by 168 h. In addition, *P. fluorescens* PV936737 displayed the largest mid-term dip in germination (61.9% at 72 h) and strong vigor decline by 168 h while *E. hormaechei* (PV936738) showed more consistent values in germination and vigor remained consistently high across all ages. Furthermore, *P. aeruginosa* PV936739 produced the highest early vigor (1202 at 24 h) and generally maintained good germination, though vigor decreased at 120 h ( Table 4).

**Table 4.** Effect of culture age on phytotoxicity of the maize seeds in presence of the test bacterial strains.

Incubation time	% germination	Germination rate	Germination time	Vigor index
***Pseudomonas aeruginosa* (PV936735)**				
24 h	80.95 ± 21.82 ^a^ ^b^	0.22 ± 0.01 ^b^	4.59 ± 0.32 ^b^	782.04 ± 444.54 ^b^ ^c^
48 h	95.24 ± 8.25 ^a^	0.22 ± 0.01 ^a^	4.52 ± 0.21 ^b^	1133.20 ± 326.08 ^a^ ^b^
72h	80.95 ± 16.50 ^a^ ^b^	0.21 ± 0.01 ^b^ ^c^	4.81 ± 0.22 ^b^	595.85 ± 52.06 ^c^
96 h	85.71 ± 14.29 ^a^ ^b^	0.20 ± 0.00 ^c^ ^d^	5.13 ± 0.30 ^a^	782.45 ± 71.91 ^b^ ^c^
120 h	66.67 ± 8.25 ^b^	0.20 ± 0.01 ^d^	5.04 ± 0.16 ^a^	386.33 ± 173.67 ^d^
144 h	95.24 ± 8.25 ^a^	0.20 ± 0.00 ^c^ ^d^	5.08 ± 0.05 ^a^	575.85 ± 42.31 ^c^ ^d^
168 h	80.95 ± 16.50 ^a^ ^b^	0.20 ± 0.00 ^c^ ^d^	5.02 ± 0.10 ^a^	189.12 ± 97.09 ^e^
***Pseudomonas aeruginosa* (PV936736)**				
24 h	71.43 ± 14.29 ^c^	0.22 ± 0.01 ^a^	4.49 ± 0.11 ^d^	659.66 ± 269.88 ^c^
48 h	90.48 ± 16.50 ^b^	0.22 ± 0.00 ^a^	4.54 ± 0.52 ^c^ ^d^	1070.41 ± 505.78 ^b^
72h	95.24 ± 8.25 ^a^	0.20 ± 0.00 ^b^	4.88 ± 0.77 ^b^	1013.81 ± 202.70 ^b^
96 h	90.48 ± 16.50 ^b^	0.20 ± 0.01 ^b^	5.08 ± 0.21 ^a^	950.14 ± 246.25 ^b^
120 h	100 ± 0 ^a^	0.20 ± 0.00 ^b^	5.10 ± 0.07 ^a^	926.19 ± 104.04 ^b^
144 h	90.48 ± 16.50 ^b^	0.20 ± 0.01 ^b^	5.05 ± 0.27 ^a^	668.23 ± 165.67 ^c^
168 h	76.19 ± 21.82 ^c^	0.21 ± 0.01 ^a^ ^b^	4.87 ± 0.14 ^b^	227.21 ± 103.59 ^d^
***Pseudomonas fluorescens* (PV936737)**				
24 h	95.24 ± 8.25 ^a^	0.22 ± 0.00 ^b^	4.48 ± 0.06 ^a^	1114.15 ± 144.06 ^b^
48 h	80.95 ± 8.25 ^a^ ^b^	0.22 ± 0.01 ^b^	4.59 ± 0.15 ^a^	868.91 ± 212.21 ^b^
72h	61.90 ± 21.82 ^c^	0.20 ± 0.03 ^c^	5.05 ± 0.66 ^c^	267.35 ± 92.25 ^c^
96 h	100 ± 0 ^a^	0.19 ± 0.01 ^c^	5.14 ± 0.22 ^c^	1007.62 ± 88.44 ^b^
120 h	85.71 ± 14.29 ^a^ ^b^	0.20 ± 0.01 ^c^	4.93 ± 0.17 ^b^ ^c^	857.62 ± 45.73 ^b^
144 h	100 ± 0 ^a^	0.20 ± 0.00 ^c^	4.99 ± 0.02 ^b^ ^c^	988.10 ± 17.98 ^b^
168 h	80.95 ± 8.25 ^a^ ^b^	0.20 ± 0.01 ^c^	5.03 ± 0.16 ^b^ ^c^	192.59 ± 82.92 ^c^
***Enterobacter hormaechei* (PV936738)**				
24 h	95.24 ± 8.25 ^a^	0.23 ± 0.00 ^b^	4.41 ± 0.09 ^a^	1116.87 ± 178.80 ^b^
48 h	95.24 ± 8.25 ^a^	0.22 ± 0.01 ^b^	4.66 ± 0.21 ^a^	1172.65 ± 366.84 ^b^
72h	85.71 ± 14.29 ^a^ ^b^	0.20 ± 0.01 ^c^	5.01 ± 0.24 ^c^	747.07 ± 297.19 ^c^
96 h	95.24 ± 8.25 ^a^	0.20 ± 0.01 ^c^	4.93 ± 0.13 ^b^ ^c^	1171.29 ± 101.69 ^b^
120 h	95.24 ± 8.25 ^a^	0.20 ± 0.00 ^c^	4.92 ± 0.10 ^b^ ^c^	858.91 ± 138.96 ^b^
144 h	95.24 ± 8.25 ^a^	0.20 ± 0.01 ^c^	5.06 ± 0.19 ^b^ ^c^	651.97 ± 201.32 ^c^
168 h	95.24 ± 8.25 ^a^	0.21 ± 0.00 ^c^	4.88 ± 0.11 ^b^ ^c^	764.29 ± 237.80 ^c^
***Pseudomonas aeruginosa* (PV936739)**				
24 h	95.24 ± 8.25 ^a^	0.23 ± 0.00 ^a^	4.36 ± 0.08 ^d^	1202.79 ± 199.50 ^b^
48 h	95.24 ± 8.25 ^a^	0.21 ± 0.01 ^b^	4.70 ± 0.12 ^c^	1029.05 ± 167.68 ^b^
72h	90.48 ± 8.25 ^a^	0.20 ± 0.01 ^c^	4.90 ± 0.17 ^b^	776.53 ± 136.25 ^c^ ^d^
96 h	95.24 ± 8.25 ^a^	0.20 ± 0.00 ^c^	4.10 ± 0.05 ^e^	1052.99 ± 157.41 ^b^
120 h	80.95 ± 16.50 ^a^	0.20 ± 0.01 ^c^	4.95 ± 0.23 ^b^	576.26 ± 162.89 ^e^
144 h	95.24 ± 8.25 ^a^	0.20 ± 0.00 ^c^	5.10 ± 0.07 ^a^	898.37 ± 133.40 ^c^ ^d^
168 h	90.48 ± 16.50 ^a^	0.20 ± 0.01 ^c^	5.00 ± 0.14 ^a^ ^b^	788.64 ± 229.45 ^c^ ^d^
Control	85.71 ± 14.29	0.21 ± 0.01	4.70 ± 0.16	963.81 ± 392.17

Within columns, values with different and same superscript letters represent differences that are significantly and not significantly different, respectively (p < 0.05).

With respect to germination, most of the treatments maintained high germination (often >80%) across culture ages. with *E. hormaechei* (PV936738) and *P. aeruginosa* (PV936739) consistently showing germination above the control. However, some declines appeared at late culture ages for *P. aeruginosa* (PV936735) and *P. fluorescens* (PV936737), suggesting mild inhibitory effects as cultures aged. In the case of germination rate, values were clustered around 0.20–0.23 across treatments, similar to the control (0.21), showing only modest influence of culture age. Slightly higher rates (0.22–0.23) were seen with very young cultures (24 h) for some of the strains; *E. hormaechei* (PV936738) and *P. aeruginosa* (PV936739), indicating a small early-stage stimulation ( Table 4).

For germination time, the recorded value for the control mean was 4.70 d, while some early cultures (24–48 h) shortened germination time to between 4.3–4.6 d (notably *P. aeruginosa* (PV936739) and *E. hormaechei* (PV936738), thus indicating mild promotion. Older cultures (96–168 h) were however observed to often prolonged germination to 5 d or more, reflecting increasing phytotoxicity as cultures aged. In addition, remarkable effects were observed with vigor index with highest values (≥1100) occurring with young cultures of *E. hormaechei* (PV936738) at 48 h and *P. aeruginosa* (PV936739) at 24 h. Most of the strains showed steep declines at 168 h, e.g., *P. aeruginosa* PV936735 (189) and *P. fluorescens* PV936737 (193), well below the control (964) ( Table 4).

***Soybean***

At the respective treatments, soybean seeds young cultures (24–48 h) followed a similar pattern as the control while older cultures (≥96 h) extended germination time and reduced vigor index. Among the strains, *P. aeruginosa* (PV936735) demonstrated age-related germinability inhibition, combining a major mid-term decline in germination% at 72 h with a strong late-stage vigor decline. For *P. aeruginosa* (PV936736), moderate germination and reduced vigor after 96 h was maintained. Also, *P. fluorescens* (PV936737) displayed high early vigor that fell below 200 at 144 h while *E. hormaechei* (PV936738) showed moderately stable germination and highest early vigor (843 at 24 h) ( Table 5).

**Table 5.** Effect of culture age on phytotoxicity of the soybean seeds in presence of the test bacterial strains.

Incubation time	% Germination	Germination Rate	Germination time	Vigor index
***Pseudomonas aeruginosa* (PV936735)**				
24 h	85.71 ± 14.29 ^a^	0.20 ± 0.01 ^b^	4.95 ± 0.18 ^a^ ^b^	493.95 ± 121.56 ^b^
48 h	61.90 ± 21.82 ^a^ ^b^	0.21 ± 0.00 ^b^	4.77 ± 0.42 ^b^	458.50 ± 357.39 ^b^
72h	57.14 ± 14.29 ^b^	0.21 ± 0.01 ^b^	4.73 ± 0.12 ^b^	215.51 ± 115.04 ^c^
96 h	85.71 ± 14.29 ^a^	0.19 ± 0.01 ^b^ ^c^	5.24 ± 0.15 ^a^	512.59 ± 148.62 ^b^
120 h	71.43 ± 0.00 ^a^ ^b^	0.20 ± 0.01 ^b^	5.01 ± 0.16 ^a^	353.74 ± 65.85 ^b^
144 h	66.67 ± 16.50 ^a^ ^b^	0.20 ± 0.01 ^b^	4.96 ± 0.15 ^a^ ^b^	336.46 ± 113.35 ^b^
168 h	76.19 ± 21.82 ^a^ ^b^	0.20 ± 0.01 ^b^ ^c^	4.91 ± 0.22 ^a^ ^b^	170.00 ± 193.06 ^c^
***Pseudomonas aeruginosa* (PV936736)**				
24 h	61.90 ± 16.50 ^a^	0.21 ± 0.00 ^a^ ^b^	4.82 ± 0.10 ^a^ ^b^	316.19 ± 229.77 ^a^ ^b^
48 h	71.43 ± 24.74 ^a^	0.21 ± 0.01 ^a^ ^b^	4.84 ± 0.22 ^a^ ^b^	388.37 ± 204.08 ^a^ ^b^
72h	61.90 ± 21.82 ^a^	0.21 ± 0.02 ^a^ ^b^	4.73 ± 0.37 ^a^ ^b^	382.59 ± 288.82 ^a^ ^b^
96 h	61.90 ± 21.82 ^a^	0.19 ± 0.02 ^a^ ^b^	5.20 ± 0.55 ^a^ ^b^	231.70 ± 226.35 ^a^ ^b^
120 h	61.90 ± 8.25 ^a^	0.20 ± 0.01 ^a^ ^b^	5.04 ± 0.22 ^a^ ^b^	205.51 ± 38.55 ^a^ ^b^
144 h	52.38 ± 21.82 ^a^	0.19 ± 0.01 ^b^	5.38 ± 0.36 ^b^	235.24 ± 201.93 ^a^ ^b^
168 h	66.67 ± 8.25 ^a^	0.20 ± 0.00 ^a^ ^b^	4.95 ± 0.12 ^a^ ^b^	139.12 ± 68.73 ^b^
***Pseudomonas fluorescens* (PV936737)**				
24 h	80.95 ± 32.99 ^a^	0.21 ± 0.00 ^a^ ^b^	4.87 ± 0.05 ^a^ ^b^	653.95 ± 417.36 ^a^ ^b^
48 h	66.67 ± 21.82 ^a^	0.21 ± 0.02 ^a^ ^b^	4.84 ± 0.36 ^a^ ^b^	424.08 ± 250.70 ^a^ ^b^
72h	76.19 ± 16.50 ^a^	0.21 ± 0.01 ^a^ ^b^	4.84 ± 0.21 ^a^ ^b^	551.90 ± 283.86 ^a^ ^b^
96 h	66.67 ± 16.50 ^a^	0.20 ± 0.11 ^a^ ^b^	5.00 ± 0.29 ^a^ ^b^	341.36 ± 104.64 ^a^ ^b^
120 h	76.19 ± 16.50 ^a^	0.19 ± 0.02 ^b^	5.24 ± 0.05 ^a^ ^b^	697.55 ± 893.88 ^a^ ^b^
144 h	57.14 ± 28.57 ^a^	0.20 ± 0.02 ^a^ ^b^	5.12 ± 0.46 ^a^ ^b^	174.56 ± 122.98 ^b^
168 h	61.90 ± 16.50 ^a^	0.21 ± 0.01 ^a^	4.72 ± 0.15 ^a^	283.20 ± 172.87 ^b^
***Enterobacter hormaechei* (PV936738)**				
24 h	76.19 ± 21.82 ^a^	0.20 ± 0.00 ^a^ ^b^	5.10 ± 0.09 ^a^ ^b^	843.49 ± 569.92 ^a^
48 h	61.90 ± 8.25 ^a^	0.21 ± 0.01 ^a^ ^b^	4.89 ± 0.32 ^a^ ^b^	342.11 ± 46.96 ^b^
72h	66.67 ± 8.25 ^a^	0.21 ± 0.01 ^a^	4.78 ± 0.21 ^a^	401.77 ± 263.56 ^b^
96 h	57.14 ± 24.74 ^a^	0.21 ± 0.01 ^a^ ^b^	4.86 ± 0.16 ^a^ ^b^	192.45 ± 181.25 ^b^
120 h	80.95 ± 21.82 ^a^	0.19 ± 0.01 ^b^	5.15 ± 0.17 ^a^ ^b^	341.84 ± 243.24 ^b^
144 h	80.95 ± 21.82 ^a^	0.21 ± 0.00 ^a^ ^b^	4.80 ± 0.02 ^a^ ^b^	424.69 ± 139.59 ^b^
168 h	61.90 ± 21.82 ^a^	0.22 ± 0.00 ^c^	4.48 ± 0.08 ^c^	193.06 ± 109.29 ^b^
***Pseudomonas aeruginosa* (PV936739)**				
24 h	66.67 ± 29.74 ^a^	0.19 ± 0.01 ^b^	5.14 ± 0.22 ^a^	474.01 ± 487.86 ^a^
48 h	61.90 ± 29.74 ^a^	0.21 ± 0.01 ^a^	4.70 ± 0.19 ^b^	459.18 ± 325.52 ^a^
72h	61.90 ± 8.25 ^a^	0.21 ± 0.01 ^a^	4.71 ± 0.13 ^b^	365.10 ± 164.69 ^a^
96 h	85.71 ± 14.29 ^a^	0.21 ± 0.00 ^a^ ^b^	4.86 ± 0.02 ^b^	620.48 ± 300.51 ^a^
120 h	85.71 ± 0.00 ^a^	0.20 ± 0.00 ^b^	4.95 ± 0.05 ^a^ ^b^	523.67 ± 110.24 ^a^
144 h	61.90 ± 8.25 ^a^	0.21 ± 0.01 ^a^ ^b^	4.82 ± 0.20 ^b^	300.75 ± 43.70 ^a^
168 h	76.19 ± 8.25 ^a^	0.22 ± 0.01 ^c^	4.57 ± 0.28 ^c^	363.95 ± 201.62 ^a^
Control	90.48 ± 16.50	0.22 ± 0.01	4.55 ± 0.18	821.90 ± 297.44

Within columns, values with different and same superscript letters represent differences that are significantly and not significantly different, respectively (p < 0.05).

With regards to germination for most treatments, moderate to high percentages (often 60–80%) was observed across culture ages, which was consistently lesser than the control value of 90%. The highest germination among the strains was observed in *P. aeruginosa* PV936739 ranging close to the control value (86% at 96–120 h). *P. aeruginosa* PV936735 showed a clear decline (57% at 72 h) before partial rise at 96 h, suggesting a temporary inhibition as the culture aged. Germination rate values were mostly between 0.19–0.21, which is close to the control (0.22), showing only a modest influence of culture age. Slightly high rates (0.20–0.21) were observed in young cultures (24–48 h) across most strains, indicating a minor early-stage stimulation ( Table 5).

The control mean for germination time was 4.55 d with younger cultures producing slightly longer values (4.7–4.9 d) except in *E. hormaechei* (PV936738) and *P. aeruginosa* PV936739 where value of 5.1 d at 24 h was recorded. In addition, cultures extended germination time beyond 5 d, notable increase in germination time were observed with increase in culture age ( Table 5).

Overall, vigor decreased considerably as cultures aged in presence of most of the strains. The vigor index of the control group recorded 821 was generally far above the vigor of the treatment ranges (200–500). Early cultures of *E. hormaechei* (PV936738) and *P. fluorescens* (PV936737) with vigor index of 843 and 654 at 24 h, respectively were however close to the control. The highest decrease was observed with *E. hormaechei* (PV936738), which dropped from about 843 at 24 h to 193 by 168 h. *P. aeruginosa* PV936735, *P. aeruginosa* PV936736 and *P. fluorescens* (PV936737) showed a similar trend with vigor reducing as cultures aged ( Table 5).

***Sorghum***

For the sorghum seeds, the germinability pattern revealed an age-dependent phytotoxic effect, with younger cultures (24–48 h) generally showing vigor index close to the control value while older cultures (≥72 h) displaying reduced vigor index. Overall, *E. hormaechei* (PV936738) showed the least phytotoxic effect while *P. aeruginosa* (PV936735) displayed the most pronounced late-age toxicity. Generally, all strains showed mid-term reduction in germinability at 72 h with strongest activity observed in presence of *P. aeruginosa* (PV936735), with a significantly lowest vigor to 119.46 and continued low values thereafter. For *P. aeruginosa* PV936736, high germination was maintained at the different culture ages, although significant drop in vigor to 129.05 at 168 h. In the case of *P. fluorescens* (PV936737), peak vigor value was recorded at early culture age (968.57 at 24 h), followed by a decline to 124.76 at 168 h. Seeds treated with *E. hormaechei* (PV936738) showed good germination across all ages and displayed the highest early vigor (1093.95 at 48 h) before a gradual decline up to 142.52 at 168 h. For *P. aeruginosa* (PV936739), consistently high germination and vigor (856.53 at 48 h) were observed with younger cultures but decreased to 128.91 at 168 h ( Table 6).

**Table 6.** Effect of culture age on phytotoxicity of the sorghum seeds in presence of the test bacterial strains.

Incubation time	% germination	Germination rate	Germination time	Vigor index
***Pseudomonas aeruginosa* (PV936735)**				
24 h	95.24 ± 8.25 ^b^	0.23 ± 0.01 ^b^	4.29 ± 0.14 ^b^	801.56 ± 24.19 ^b^
48 h	85.71 ± 14.29 ^b^	0.25 ± 0.00 ^a^	4.06 ± 0.06 ^b^	531.70 ± 206.66 ^c^
72h	66.67 ± 8.25 ^c^	0.23 ± 0.00 ^b^	4.40 ± 0.09 ^b^	119.46 ± 55.44 ^e^
96 h	90.47 ± 8.25 ^b^	0.21 ± 0.01 ^c^	4.81 ± 0.18 ^a^	526.53 ± 148.01 ^c^
120 h	80.95 ± 8.25 ^b^ ^c^	0.20 ± 0.02 ^c^	5.07 ± 0.38 ^a^	273.95 ± 76.53 ^d^
144 h	100 ± 0.00 ^a^	0.20 ± 0.00 ^c^	4.99 ± 0.05 ^a^	147.14 ± 43.78 ^e^
168 h	76.19 ± 8.25 ^b^ ^c^	0.22 ± 0.00 ^b^ ^c^	4.63 ± 0.08 ^a^ ^b^	58.70 ± 22.47 ^e^
***Pseudomonas aeruginosa* (PV936736)**				
24 h	90.48 ± 8.25 ^a^	0.24 ± 0.00 ^a^	4.14 ± 0.07 ^c^	819.66 ± 33.10 ^d^
48 h	80.95 ± 21.82 ^a^	0.21 ± 0.00 ^b^	4.68 ± 0.05 ^b^	702.72 ± 322.28 ^d^
72h	85.71 ± 14.29 ^a^	0.23 ± 0.01 ^a^ ^b^	4.29 ± 0.16 ^c^	290.20 ± 60.42 ^c^
96 h	95.24 ± 8.25 ^a^	0.23 ± 0.01 ^a^ ^b^	4.38 ± 0.11 ^c^	641.29 ± 28.47 ^c^
120 h	80.95 ± 8.25 ^a^	0.20 ± 0.01 ^c^	4.92 ± 0.17 ^a^	266.94 ± 117.79 ^b^ ^c^
144 h	90.48 ± 16.20 ^a^	0.20 ± 0.00 ^c^	5.03 ± 0.07 ^a^	134.69 ± 166.10 ^a^ ^b^
168 h	100.00 ± 0.00 ^a^	0.22 ± 0.00 ^b^	4.61 ± 0.06 ^b^	129.05 ± 113.52 ^a^
***Pseudomonas fluorescens* (PV936737)**				
24 h	100 ± 0 ^a^	0.23 ± 0.00 ^b^ ^c^	4.29 ± 0.07 ^a^ ^b^	968.57 ± 63.58 ^b^ ^c^
48 h	80.95 ± 21.82 ^b^	0.24 ± 0.01 ^b^	4.23 ± 0.22 ^a^ ^b^	740.20 ± 369.99 ^c^
72h	90.48 ± 8.25 ^a^ ^b^	0.21 ± 0.01 ^c^ ^d^	4.76 ± 0.27 ^a^	179.93 ± 118.75 ^d^
96 h	90.48 ± 8.25 ^a^ ^b^	0.22 ± 0.00 ^c^	4.51 ± 0.07 ^a^ ^b^	469.39 ± 99.89 ^c^ ^d^
120 h	90.48 ± 8.25 ^a^ ^b^	0.20 ± 0.01 ^d^	5.01 ± 0.25 ^b^	432.18 ± 170.88 ^c^ ^d^
144 h	90.48 ± 8.25 ^a^ ^b^	0.20 ± 0.00 ^d^	4.93 ± 0.06 ^b^	155.71 ± 75.50 ^d^
168 h	100 ± 0 ^a^	0.22 ± 0.00 ^c^	4.63 ± 0.06 ^a^ ^b^	124.76 ± 101.64 ^d^
***Enterobacter hormaechei* (PV936738)**				
24 h	90.48 ± 16.50 ^a^	0.23 ± 0.01 ^a^	4.29 ± 0.10 ^b^	732.86 ± 307.48 ^b^
48 h	95.24 ± 8.25 ^a^	0.24 ± 0.01 ^a^	4.25 ± 0.11 ^b^	1093.95 ± 236.94 ^a^
72h	85.71 ± 14.29 ^a^	0.22 ± 0.02 ^b^	4.48 ± 0.34 ^b^	216.80 ± 77.08 ^c^
96 h	85.71 ± 0.00 ^a^	0.23 ± 0.02 ^b^	4.46 ± 0.31 ^b^	380.82 ± 128.56 ^c^
120 h	76.19 ± 16.50 ^a^	0.20 ± 0.01 ^c^	4.96 ± 0.15 ^a^	300.41 ± 106.03 ^c^
144 h	95.24 ± 8.25 ^a^	0.20 ± 0.00 ^c^	4.93 ± 0.07 ^a^	185.65 ± 70.84 ^c^
168 h	85.71 ± 14.29 ^a^	0.22 ± 0.00 ^b^	4.54 ± 0.04 ^b^	142.52 ± 122.90 ^c^
***Pseudomonas aeruginosa* (PV936739)**				
24 h	85.71 ± 14.29 ^a^	0.24 ± 0.01 ^a^	4.16 ± 0.16 ^c^	753.67 ± 275.27 ^b^
48 h	85.71 ± 14.29 ^a^	0.23 ± 0.01 ^a^ ^b^	4.33 ± 0.16 ^c^	856.53 ± 352.15 ^b^
72h	85.71 ± 14.29 ^a^	0.22 ± 0.02 ^c^	4.66 ± 0.49 ^b^	205.85 ± 121.25 ^c^
96 h	100.00 ± 0.00 ^a^	0.22 ± 0.00 ^b^ ^c^	4.48 ± 0.96 ^b^ ^c^	585.71 ± 96.50 ^b^
120 h	90.48 ± 16.50 ^a^	0.21 ± 0.00 ^d^	4.76 ± 0.39 ^a^	459.86 ± 208.06 ^c^
144 h	95.24 ± 8.25 ^a^	0.21 ± 0.00 ^d^	4.66 ± 0.75 ^b^	199.80 ± 108.38 ^c^
168 h	90.48 ± 8.25 ^a^	0.22 ± 0.00 ^b^ ^c^	4.55 ± 0.62 ^b^ ^c^	128.91 ± 31.22 ^c^
Control	95.24 ** ± 8.25**	0.24 ** ± 0.01**	4.21 ** ± 0.18**	818.57 ** ± 308.57**

Within columns, values with different and same superscript letters represent differences that are significantly and not significantly different, respectively (p < 0.05).

With respect to germination, most treatments maintained high percentages (≥80%) with all treatments, except *P. aeruginosa* PV936735 (67% at 72 h and 76% at 168 h) consistently in close range or exceeded the control value of 95%. Germination rate values were observed to cluster around 0.20–0.24 d, which was similar to the control (0.24). However, slightly high rates (0.23–0.25 d) occurred in young cultures (24–48 h) of *P. aeruginosa* (PV936735, PV936737 and PV936739), and *E. hormaechei* (PV936738) ( Table 6).

For germination time, the recorded value for the control mean was 4.21 d and remained in the 4.1–4.3 d range for most strains at 24–48 h, but increased to 4.8–5.1 d at 96–144 h for *P. aeruginosa* (PV936735, PV936736), and *E. hormaechei* (PV936738), indicating the onset of age-related inhibition ( Table 6).

The vigor index showed the most prominent changes with control vigor index value of 818.57, while early cultures (24–48 h) of all strains showed values close to or exceeding the control value except *P. aeruginosa* (PV936735) (531.70 at 48 h). All strains however showed severe decline at 168 h with the lowest values seen in *P. aeruginosa* (PV936735) (58.70) and *P. fluorescens* (PV936737) (124.76), confirming accumulation of phytotoxic metabolites as cultures aged ( Table 6).

***Cucumber***

For the cucumber seeds, at the respective inoculum ages, younger cultures (24–48 h) were observed to follow similar trend as the control, while older cultures (≥72 h) showed decrease in germination rate (≥96 h) and showed a slight increase in germination time and modest reductions in vigor. Among the strains, *P. aeruginosa* PV936735 showed the highest early vigor (24 h) and highest inhibitory effect at 168 h (843.74). *P. aeruginosa* (PV936736) maintained a moderately high germination and vigor up to the oldest culture age (168 h) despite a temporary extension of germination time at 96 h (5.44 d). *P. fluorescens* (PV936737) produced the highest vigor index (1312.38) and maintained a vigor value higher than the control even at the oldest culture age. *E. hormaechei* (PV936738) demonstrated steady high germination and vigor throughout all ages except at 72 h and 168 h. *P. aeruginosa* (PV936739) maintained a steady high germination and a late-age decline at 168 h ( Table 7).

**Table 7.** Effect of culture age on phytotoxicity of the cucumber seeds in presence of the test bacterial strains.

Incubation time	% Germination	Germination rate	Germination time	Vigor index
***Pseudomonas aeruginosa* (PV936735)**				
24 h	100 ± 0.00 ^a^	0.23 ± 0.00 ^a^	4.27 ± 0.07 ^a^	1210.95 ± 170.39 ^a^
48 h	95.24 ± 8.25 ^a^	0.23 ± 0.00 ^a^	4.44 ± 0.07 ^a^	931.70 ± 322.34 ^a^
72h	100 ± 0.00 ^a^	0.22 ± 0.01 ^b^	4.56 ± 0.24 ^b^	1221.43 ± 23.60 ^a^
96 h	90.48 ± 16.50 ^a^	0.21 ± 0.01 ^c^	4.87 ± 0.16 ^c^	1064.22 ± 326.72 ^a^
120 h	100 ± 0.00 ^a^	0.20 ± 0.01 ^d^	5.12 ± 0.21 ^d^	1169.05 ± 31.70 ^a^
144 h	100 ± 0.00 ^a^	0.20 ± 0.00 ^d^	4.95 ± 0.06 ^d^	1182.38 ± 95.86 ^a^
168 h	90.48 ± 8.25 ^a^	0.21 ± 0.01 ^c^	4.69 ± 0.16 ^b^ ^c^	843.74 ± 95.48 ^b^
***Pseudomonas aeruginosa* (PV936736)**				
24 h	95.24 ± 8.25 ^a^	0.23 ± 0.01 ^a^	4.34 ± 0.12 ^a^ ^b^	1037.48 ± 289.03 ^a^
48 h	100 ± 0.00 ^a^	0.22 ± 0.01 ^a^	4.59 ± 0.21 ^a^ ^b^	1057.14 ± 188.88 ^a^
72h	100 ± 0.00 ^a^	0.22 ± 0.00 ^a^	4.59 ± 0.09 ^a^ ^b^	1205.24 ± 148.03 ^a^
96 h	95.24 ± 8.25 ^a^	0.18 ± 0.02 ^d^	5.44 ± 0.47 ^d^	1054.56 ± 179.51 ^a^
120 h	100 ± 0.00 ^a^	0.20 ± 0.01 ^c^	4.99 ± 0.29 ^c^	1153.33 ± 124.10 ^a^
144 h	100 ± 0.00 ^a^	0.20 ± 0.00 ^c^	4.92 ± 0.09 ^c^	1230.00 ± 86.65 ^a^
168 h	95.24 ± 8.25 ^a^	0.22 ± 0.00 ^b^	4.61 ± 0.07 ^a^ ^b^	1045.24 ± 149.62 ^a^
***Pseudomonas fluorescens* (PV936737)**				
24 h	95.24 ± 8.25 ^a^	0.22 ± 0.01 ^a^	4.48 ± 0.17 ^b^	1076.60 ± 230.06 ^a^
48 h	100 ± 0.00 ^a^	0.23 ± 0.01 ^a^	4.41 ± 0.17 ^b^	1041.43 ± 187.63 ^a^
72h	100 ± 0.00 ^a^	0.23 ± 0.00 ^a^	4.37 ± 0.05 ^b^	1312.38 ± 37.37 ^a^
96 h	95.24 ± 8.25 ^a^	0.20 ± 0.01 ^c^	4.95 ± 0.22 ^a^	1015.78 ± 220.16 ^a^
120 h	95.24 ± 8.25 ^a^	0.20 ± 0.00 ^c^	4.98 ± 0.11 ^a^	1077.89 ± 255.89 ^a^
144 h	100 ± 0.00 ^a^	0.20 ± 0.00 ^c^	4.90 ± 0.02 ^a^	1210.00 ± 105.74 ^a^
168 h	100 ± 0.00 ^a^	0.22 ± 0.00 ^b^	4.52 ± 0.04 ^b^	1180.95 ± 32.59 ^a^
***Enterobacter hormaechei* (PV936738)**				
24 h	100 ± 0.00 ^a^	0.23 ± 0.00 ^a^	4.32 ± 0.05 ^a^	1186.67 ± 111.14 ^a^
48 h	100 ± 0.00 ^a^	0.23 ± 0.00 ^a^	4.38 ± 0.04 ^a^	1123.81 ± 75.68 ^a^
72h	90.46 ± 16.50 ^a^	0.22 ± 0.01 ^a^	4.51 ± 0.24 ^a^	957.96 ± 433.10 ^a^
96 h	100 ± 0.00 ^a^	0.21 ± 0.01 ^c^	4.79 ± 0.16 ^c^	1192.86 ± 36.62 ^a^
120 h	100 ± 0.00 ^a^	0.20 ± 0.00 ^d^	4.90 ± 0.08 ^d^	1047.14 ± 132.81 ^a^
144 h	100 ± 0.00 ^a^	0.21 ± 0.00 ^d^	4.88 ± 0.04 ^d^	1154.29 ± 69.68 ^a^
168 h	100 ± 0.00 ^a^	0.22 ± 0.00 ^b^	4.58 ± 0.08 ^b^	991.90 ± 184.03 ^a^
***Pseudomonas aeruginosa* (PV936739)**				
24 h	90.48 ± 8.25 ^b^	0.23 ± 0.00 ^a^	4.39 ± 0.05 ^a^ ^b^	1024.08 ± 184.90 ^a^ ^b^
48 h	95.24 ± 8.25 ^a^ ^b^	0.23 ± 0.00 ^a^	4.34 ± 0.08 ^a^	994.76 ± 230.58 ^a^ ^b^
72h	100 ± 0.00 ^a^	0.22 ± 0.01 ^a^	4.45 ± 0.11 ^a^	1152.86 ± 69.34 ^a^
96 h	100 ± 0.00 ^a^	0.22 ± 0.00 ^a^	4.51 ± 0.06 ^a^	1253.33 ± 52.11 ^a^
120 h	100 ± 0.00 ^a^	0.20 ± 0.00 ^c^	5.05 ± 0.08 ^c^	1091.90 ± 179.20 ^a^ ^b^
144 h	100 ± 0.00 ^a^	0.20 ± 0.00 ^c^	4.88 ± 0.04 ^b^	1245.71 ± 91.83 ^a^
168 h	100 ± 0.00 ^a^	0.22 ± 0.00 ^b^	4.62 ± 0.07 ^a^	854.29 ± 120.10 ^b^
Control	90.48 ** ± **8.25	0.23 ** ± **0.01	4.34 ** ± **0.13	1007.21 ** ± **179.97

Within columns, values with different and same superscript letters represent differences that are significantly and not significantly different, respectively (p < 0.05).

With respect to germination, all treatments maintained very high percentages (mostly 90–100%) across all culture ages, consistently having the same value or exceeding the control (90.48%). In the case of germination rate, values majorly around 0.20–0.23, close to the control (0.23), showing reduced influence of culture age. High rates (0.23) which is similar to the control value were seen at 24–48 h for most strains, indicating a mild early-stage stimulation that reduced slightly (0.20) at 120 h except for *P. aeruginosa* (PV936736) which showed the least rate at 96 h (0.18) ( Table 7).

For germination time **,** the control mean was 4.34 d. Young cultures (24–48 h) maintained similar values (4.3–4.4 d), while older cultures showed mild extended germination time. Significant increases in germination time were observed to occur with *P. aeruginosa* (PV936736) (5.44 d at 96 h), *P. aeruginosa* (PV936735) (5.12 d at 120 h) and *P. aeruginosa* (PV936739) (5.05 d at 120 h), as cultures aged, especially within 96–144 h across all strains ( Table 7).

Vigor index for the control was 1007.21. Highest value (≥1200) occurred with young cultures of *P. fluorescens* (PV936737) (1312.38 at 72 h), *P. aeruginosa* (PV936735) (1210.95 at 24 h and 1221.43 at 72 h) and *P. aeruginosa* (PV936736) (1205.24 at 72 h). Some older cultures (144 h) also showed high values (≥1200) as seen in *P. aeruginosa* (PV936736) (1230.00), *P. fluorescens* (PV936737) (1210.00) and *P. aeruginosa* (PV936739) (1245.71) ( Table 7).

**3.3.2 Effect of stepping duration**

***Cowpea***

At the respective steeping durations, no consistent pattern of increases or decreases were observed for the germinability parameters investigated at the different steeping times. This observation was irrespective of the test bacterial strains. In presence of the bacterial treatments, germination that ranged from 80-90%, 47-100%, 76-95%, 61-100% and 61-95% were observed in presence of *P. aeruginosa* (PV936735), *P. aeruginosa* (PV936736), *P. fluorescens* (PV936737), *E. hormaechei* (PV936738) and *P. aeruginosa* (PV936739), respectively. Overall, shorter steeping time (1–2 h) showed minute effect on the vigor index ( Table 8).

**Table 8.** Effect of steeping time on phytotoxicity of the cowpea seeds in presence of the test bacterial strains.

Steeping time	% germination	Germination rate	Germination time	Vigor index
***Pseudomonas aeruginosa* (PV936735)**				
1 h	85.71 ± 14.29 ^a^	0.22 ± 0.00 ^a^	4.49 ± 0.09 ^a^	1162.99 ± 487.22 ^a^
2 h	80.95 ± 21.82 ^a^	0.22 ± 0.00 ^a^	4.49 ± 0.09 ^a^	1162.99 ± 487.22 ^a^
3 h	76.19 ± 8.25 ^a^	0.23 ± 0.01 ^a^	4.30 ± 0.14 ^a^	1425.17 ± 735.09 ^a^
4 h	80.95 ± 8.25 ^a^	0.22 ± 0.01 ^a^	4.31 ± 0.20 ^a^	958.57 ± 285.50 ^a^
5 h	90.48 ± 8.25 ^a^	0.23 ± 0.01 ^a^	4.52 ± 0.20 ^a^	1174.22 ± 438.54 ^a^
***Pseudomonas aeruginosa* (PV936736)**				
1 h	100 ± 0.00 ^a^	0.23 ± 0.00 ^a^	4.28 ± 0.03 ^c^	1882.86 ± 248.67 ^a^
2 h	66.67 ± 8.25 ^b^	0.22 ± 0.00 ^a^ ^b^	4.46 ± 0.10 ^b^	672.72 ± 150.99 ^c^
3 h	76.19 ± 16.50 ^b^	0.21 ± 0.00 ^b^	4.72 ± 0.07 ^a^	797.96 ± 319.92 ^b^ ^c^
4 h	95.24 ± 8.25 ^a^ ^b^	0.21 ± 0.02 ^b^	4.77 ± 0.42 ^a^	1364.97 ± 521.34 ^a^ ^b^
5 h	47.62 ± 32.99 ^c^	0.21 ± 0.01 ^b^	4.74 ± 0.21 ^a^	392.24 ± 623.25 ^c^
***Pseudomonas fluorescens* (PV936737)**				
1 h	80.95 ± 8.25 ^a^	0.22 ± 0.02 ^a^	4.49 ± 0.36 ^a^	1167.55 ± 476.85 ^a^
2 h	85.71 ± 0.00 ^a^	0.23 ± 0.00 ^a^	4.39 ± 0.08 ^a^	1322.86 ± 273.34 ^a^
3 h	76.19 ± 21.82 ^a^	0.22 ± 0.00 ^a^	4.47 ± 0.08 ^a^	1269.59 ± 876.44 ^a^
4 h	85.71 ± 14.29 ^a^	0.22 ± 0.01 ^a^	4.58 ± 0.24 ^a^	996.87 ± 606.48 ^a^
5 h	95.24 ± 8.25 ^a^	0.22 ± 0.01 ^a^	4.57 ± 0.12 ^a^	1500.20 ± 759.03 ^a^
***Enterobacter hormaechei* (PV936738)**				
1 h	61.90 ± 16.50 ^c^	0.23 ± 0.01 ^b^	4.43 ± 0.11 ^b^	819.12 ± 302.94 ^c^
2 h	95.24 ± 8.25 ^a^	0.23 ± 0.00 ^a^	4.26 ± 0.08 ^c^	1965.24 ± 312.58 ^a^
3 h	90.48 ± 16.50 ^a^ ^b^	0.22 ± 0.01 ^b^	4.48 ± 0.20 ^b^	986.94 ± 548.29 ^b^ ^c^
4 h	100 ± 0.00 ^a^	0.21 ± 0.00 ^c^	4.67 ± 0.06 ^a^	1270.95 ± 533.64 ^b^
5 h	80.95 ± 8.25 ^b^	0.23 ± 0.00 ^a^ ^b^	4.35 ± 0.06 ^c^	1165.03 ± 241.41 ^b^
***Pseudomonas aeruginosa* (PV936739)**				
1 h	71.43 ± 14.29 ^a^ ^b^	0.23 ± 0.01 ^a^	4.33 ± 0.19 ^a^	1015.78 ± 380.38 ^a^ ^b^
2 h	85.71 ± 0.00 ^a^ ^b^	0.23 ± 0.00 ^a^	4.37 ± 0.05 ^a^	1272.65 ± 159.64 ^a^ ^b^
3 h	61.90 ± 32.99 ^b^	0.23 ± 0.01 ^a^	4.44 ± 0.21 ^a^	721.56 ± 613.53 ^b^
4 h	95.24 ± 8.25 ^a^	0.23 ± 0.00 ^a^	4.35 ± 0.06 ^a^	1623.74 ± 276.33 ^a^
5 h	95.24 ± 8.25 ^a^	0.23 ± 0.00 ^a^	4.38 ± 0.07 ^a^	1601.16 ± 582.30 ^a^
**Control**				
1 h	95.24 ± 8.25 ^a^	0.23 ± 0.01 ^a^	4.35 ± 0.17 ^a^	1583.20 ± 532.71 ^b^
2 h	80.95 ± 8.25 ^a^ ^b^	0.23 ± 0.01 ^a^	4.32 ± 0.13 ^a^	1205.17 ± 101.05 ^b^
3 h	71.43 ± 14.29 ^c^	0.24 ± 0.01 ^a^	4.13 ± 0.12 ^a^	763.57 ± 178.21 ^c^
4 h	100 ± 0 ^a^	0.23 ± 0.01 ^a^	4.27 ± 0.10 ^a^	2289.05 ± 137.50 ^a^
5 h	95.24 ± 8.25 ^a^	0.23 ± 0.01 ^a^	4.30 ± 0.13 ^a^	1078.98 ± 543.95 ^b^

Within columns, values with different and same superscript letters represent differences that are significantly and not significantly different, respectively (p < 0.05).

Across the treatments, no significant difference between germination rates at the different steeping durations were observed. Overall, germination rate that ranged from 0.21-0.23 d ^−1^ was observed, which was similar to the control ( Table 8).

For germination time, values that ranged from 4.30-4.52 [ *P. aeruginosa* (PV936735)] 4.28-4.77 d *P. aeruginosa* [(PV936736)], 4.39-4.58 d [ *P. fluorescens* (PV936737)], 4.26-4.67 d [ *E. hormaechei* (PV936738)] and 4.33-4.44 d [ *P. aeruginosa* (PV936739)] were observed. Longer germination time was observed at longer steeping durations (4-5 h). This observation was also irrespective of the test bacterial strains. Germination time extension was observed for *P. aeruginosa* PV936736 at 3–5 h (4.72–4.77 d) and a slight reduction for *E. hormaechei* at 2 h (4.26 d).

In the case of vigor, significantly lowest values of 958.57, 392, 996.87, 819.12 and 721.56 were observed in the presence of *P. aeruginosa* (PV936735), *P. aeruginosa* (PV936736), *P. fluorescens* (PV936737), *E. hormaechei* (PV936738) and *P. aeruginosa* (PV936739), at steeping durations of 4 h, 5 h, 4 h, 1 h and 3 h, respectively. *E. hormaechei* showed the highest vigor of 1965.24 at 2 h before declining to 1165.03 at 5 h while *P. aeruginosa* PV936736 showed a high vigor (1882.86 at 1 h) before declining rapidly to 392.24 at 5 h. For *P. aeruginosa* PV936739, a remarkable decline in vigor at 2 h from 1272.65 to 721.56 at 3 h was observed ( Table 8).

***Maize***

At the different steeping durations, there was no observed pattern of increases or decreases in germination of the maize seeds at the respective treatments and control setup. This observation was irrespective of the treatments. For the *P. aeruginosa* (PV936735) treatment, highest germination was observed 4 h (85.71%), with decreases to between 61-67% at 3h and 5h. For the *P. aeruginosa* (PV936736) treated seeds, increase in germination was observed from 71% to 90.48% at 5 h while and *P. fluorescens* (PV936737) showed high overall germination (80-100%) among the isolates, with highest value recorded at 3 h steeping time. Similarly, *E. hormaechei* (PV936738) treatments showed stability between 71-85% at the respective treatments while *P. aeruginosa* (PV936739) showed lower germination at lower steeping time, increasing to 85% by 3-5h ( Table 9).

**Table 9.** Effect of steeping time on phytotoxicity of the maize seeds in presence of the test bacterial strains.

Steeping time	% germination	Germination rate	Germination time	Vigor index
***Pseudomonas aeruginosa* (PV936735)**				
1 h	66.67 ± 8.25 ^a^	0.21 ± 0.01 ^a^	4.67 ± 0.22 ^a^	338.44 ± 189.71 ^a^
2 h	71.43 ± 28.57 ^a^	0.21 ± 0.01 ^a^	4.83 ± 0.20 ^a^	549.32 ± 527.07 ^a^
3 h	61.90 ± 16.50 ^a^	0.21 ± 0.00 ^a^	4.75 ± 0.10 ^a^	318.91 ± 112.87 ^a^
4 h	85.71 ± 14.29 ^a^	0.21 ± 0.00 ^a^	4.75 ± 0.03 ^a^	681.56 ± 296.79 ^a^
5 h	66.67 ± 16.50 ^a^	0.20 ± 0.00 ^a^	4.89 ± 0.10 ^a^	317.96 ± 145.94 ^a^
***Pseudomonas aeruginosa* (PV936736)**				
1 h	71.43 ± 14.29 ^b^	0.20 ± 0.00 ^c^	5.07 ± 0.06 ^a^	411.43 ± 103.63 ^d^
2 h	76.19 ± 8.25 ^b^	0.21 ± 0.00 ^a^	4.66 ± 0.07 ^b^	486.26 ± 31.47 ^c^
3 h	80.95 ± 8.25 ^b^	0.21 ± 0.01 ^b^	4.78 ± 0.20 ^b^	638.64 ± 123.51 ^b^
4 h	76.19 ± 8.25 ^b^	0.21 ± 0.00 ^a^ ^b^	4.86 ± 0.10 ^b^	552.04 ± 41.46 ^c^
5 h	90.48 ± 8.25 ^a^	0.21 ± 0.00 ^b^	4.78 ± 0.09 ^b^	765.65 ± 132.79 ^a^
***Pseudomonas fluorescens* (PV936737)**				
1 h	85.71 ± 14.29 ^a^	0.21 ± 0.00 ^b^	4.79 ± 0.03 ^a^	639.73 ± 470.75 ^a^
2 h	80.95 ± 8.25 ^a^	0.21 ± 0.01 ^b^	4.73 ± 0.17 ^a^	998.03 ± 846.53 ^a^
3 h	100 ± 0.00 ^a^	0.21 ± 0.00 ^b^	4.74 ± 0.08 ^a^	899.52 ± 51.46 ^a^
4 h	85.71 ± 14.29 ^a^	0.21 ± 0.00 ^b^	4.76 ± 0.03 ^a^	750.82 ± 215.26 ^a^
5 h	85.71 ± 14.29 ^a^	0.20 ± 0.02 ^a^	5.10 ± 0.40 ^b^	428.71 ± 148.36 ^a^
***Enterobacter hormaechei* (PV936738)**				
1 h	71.43 ± 14.29 ^a^	0.21 ± 0.01 ^a^ ^b^	4.70 ± 0.13 ^a^ ^b^	469.18 ± 200.53 ^a^
2 h	85.71 ± 0.00 ^a^	0.21 ± 0.00 ^a^ ^b^	4.72 ± 0.08 ^a^ ^b^	555.10 ± 126.36 ^a^
3 h	85.71 ± 14.29 ^a^	0.21 ± 0.01 ^a^ ^b^	4.80 ± 0.22 ^a^ ^b^	648.03 ± 312.94 ^a^
4 h	85.71 ± 0.00 ^a^	0.21 ± 0.00 ^a^ ^b^	4.67 ± 0.05 ^a^ ^b^	806.94 ± 105.13 ^b^
5 h	71.43 ± 14.29 ^a^	0.20 ± 0.00 ^a^	4.96 ± 0.11 ^b^	414.90 ± 182.64 ^a^
***Pseudomonas aeruginosa* (PV936739)**				
1 h	57.14 ± 14.29 ^a^	0.21 ± 0.02 ^a^	4.82 ± 0.47 ^a^	294.69 ± 170.33 ^a^
2 h	76.19 ± 8.25 ^a^ ^b^	0.21 ± 0.01 ^a^	4.74 ± 0.21 ^a^	669.80 ± 171.09 ^a^ ^b^
3 h	85.71 ± 14.29 ^b^	0.21 ± 0.00 ^a^	4.74 ± 0.04 ^a^	818.57 ± 288.63 ^b^
4 h	80.95 ± 8.25 ^b^	0.22 ± 0.00 ^a^	4.64 ± 0.03 ^a^	571.56 ± 86.37 ^a^ ^b^
5 h	85.71 ± 14.29 ^b^	0.21 ± 0.00 ^a^	4.74 ± 0.08 ^a^	725.17 ± 297.25 ^b^
**Control**				
1 h	90.48 ± 16.50 ^a^	0.20 ± 0.01 ^b^	4.93 ± 0.21 ^a^	547.07 ± 272.38 ^a^
2 h	80.95 ± 8.25 ^a^	0.22 ± 0.01 ^a^	4.56 ± 0.17 ^b^	759.12 ± 78.30 ^a^
3 h	85.71 ± 14.29 ^a^	0.21 ± 0.01 ^a^ ^b^	4.68 ± 0.12 ^a^ ^b^	738.50 ± 351.55 ^a^
4 h	95.24 ± 8.25 ^a^	0.21 ± 0.01 ^a^ ^b^	4.78 ± 0.15 ^a^ ^b^	912.99 ± 385.08 ^a^
5 h	76.19 ± 29.74 ^a^	0.21 ± 0.01 ^b^	4.88 ± 0.20 ^a^	500.68 ± 298.78 ^a^

Within columns, values with different and same superscript letters represent differences that are significantly and not significantly different, respectively (p < 0.05).

Germination rate of the seeds remained stable between 0.20-0.22 d-1 for all the bacterial treatment and control seeds with no observed pattern at the respective steeping times. In addition, germination time showed stability for all the treatment groups and control, varying from 4.56 to 5.10 d. However, slight variations were observed depending on the treatment and time. Although some decreases were observed in presence of some of the bacterial treatments, no consistency was observed ( Table 9).

With respect to vigor index, wide variations were observed at the different steeping times in treatments with *P. aeruginosa* (PV936735), with highest value at 4h (681) and lowest at 3h and 5h (318). A general increasing trend to 765.65 (5 h), 807 (4 h) and 819 (3 h) was however observed for treatments with *P. aeruginosa* (PV936736) and *E. hormaechei* (PV936738) and *P. aeruginosa* (PV936739), respectively while *P. fluorescens* (PV936737) showed high vigor index overall, with highest at 2h (998), before declining at 5h. For the control seeds, general increase in vigor with increase in steeping time was observed till 4 h (913) before a drop at 5 h ( Table 9).

***Soybean***

For soybean seeds at different steeping durations, no obvious germinability pattern was observed for all treatments and control setup. The germination percentages observed for bacterial treatments ranges from 71-90%, 76-86%, 76-90%, 81-90% and 81-95% respectively for *P. aeruginosa* (PV936735), *P. aeruginosa* (PV936736), *P. fluorescens* (PV936737), *E. hormaechei* (PV936738) and *P. aeruginosa* (PV936739). However, *P. aeruginosa* PV936735 showed a steady downward trend from 90.48% at 1 h to 71.43% at 5 h ( Table 10).

**Table 10.** Effect of steeping time on phytotoxicity of the soybean seeds in presence of the test bacterial strains.

Steeping time	% germination	Germination rate	Germination time	Vigor index
***Pseudomonas aeruginosa* (PV936735)**				
1 h	90.48 ± 8.25 ^a^	0.23 ± 0.00 ^a^ ^b^	4.38 ± 0.06 ^a^	999.66 ± 258.76 ^a^
2 h	85.71 ± 14.29 ^a^	0.23 ± 0.01 ^a^ ^b^	4.44 ± 0.13 ^a^ ^b^	945.44 ± 314.54 ^a^
3 h	80.95 ± 8.25 ^a^	0.22 ± 0.00 ^b^	4.60 ± 0.02 ^b^	816.53 ± 223.58 ^a^
4 h	76.19 ± 21.82 ^a^	0.23 ± 0.00 ^a^	4.39 ± 0.03 ^a^	1065.65 ± 710.27 ^a^
5 h	71.43 ± 14.29 ^a^	0.22 ± 0.01 ^a^ ^b^	4.50 ± 0.11 ^a^ ^b^	821.09 ± 469.66 ^a^
***Pseudomonas aeruginosa* (PV936736)**				
1 h	80.95 ± 8.25 ^a^	0.22 ± 0.01 ^a^	4.52 ± 0.22 ^a^	1386.60 ± 1177.37 ^a^
2 h	76.19 ± 16.50 ^a^	0.22 ± 0.00 ^a^	4.49 ± 0.06 ^a^	804.63 ± 411.10 ^a^
3 h	80.95 ± 8.25 ^a^	0.23 ± 0.01 ^a^	4.44 ± 0.21 ^a^	1022.38 ± 294.48 ^a^
4 h	80.95 ± 8.25 ^a^	0.22 ± 0.00 ^a^	4.56 ± 0.04 ^a^	945.78 ± 249.83 ^a^
5 h	85.71 ± 14.29 ^a^	0.21 ± 0.01 ^a^	4.66 ± 0.22 ^a^	1078.71 ± 532.60 ^a^
***Pseudomonas fluorescens* (PV936737)**				
1 h	90.48 ± 16.50 ^a^	0.22 ± 0.01 ^a^	4.60 ± 0.14 ^a^	627.62 ± 191.42 ^a^
2 h	85.71 ± 0.00 ^a^	0.22 ± 0.01 ^a^	4.47 ± 0.10 ^a^	903.67 ± 315.38 ^a^
3 h	80.95 ± 21.82 ^a^	0.22 ± 0.00 ^a^	4.51 ± 0.10 ^a^	1069.86 ± 491.06 ^a^
4 h	76.19 ± 16.50 ^a^	0.23 ± 0.01 ^a^	4.42 ± 0.24 ^a^	981.36 ± 509.56 ^a^
5 h	80.95 ± 8.25 ^a^	0.22 ± 0.01 ^a^	4.60 ± 0.16 ^a^	941.97 ± 68.86 ^a^
***Enterobacter hormaechei* (PV936738)**				
1 h	85.71 ± 14.29 ^a^	0.22 ± 0.00 ^a^	4.57 ± 0.05 ^a^	780.48 ± 170.23 ^a^
2 h	85.71 ± 14.29 ^a^	0.22 ± 0.01 ^a^	4.49 ± 0.19 ^a^	1048.16 ± 472.79 ^a^
3 h	80.95 ± 8.25 ^a^	0.23 ± 0.00 ^a^	4.31 ± 0.08 ^a^	1172.31 ± 293.69 ^a^
4 h	85.71 ± 14.29 ^a^	0.22 ± 0.01 ^a^	4.64 ± 0.37 ^a^	1054.35 ± 411.32 ^a^
5 h	90.48 ± 16.50 ^a^	0.22 ± 0.01 ^a^	4.55 ± 0.18 ^a^	1247.62 ± 485.82 ^a^
***Pseudomonas aeruginosa* (PV936739)**				
1 h	85.71 ± 14.29 ^a^	0.23 ± 0.00 ^b^	4.30 ± 0.07 ^b^	1062.72 ± 346.97 ^a^
2 h	80.95 ± 21.82 ^a^	0.22 ± 0.00 ^a^ ^b^	4.52 ± 0.05 ^a^	935.51 ± 467.58 ^a^
3 h	95.24 ± 8.25 ^a^	0.22 ± 0.00 ^a^ ^b^	4.50 ± 0.04 ^a^ ^b^	133.88 ± 245.32 ^a^
4 h	85.71 ± 14.29 ^a^	0.22 ± 0.01 ^a^ ^b^	4.54 ± 0.12 ^a^	957.76 ± 506.22 ^a^
5 h	95.24 ± 8.25 ^a^	0.22 ± 0.01 ^a^ ^b^	4.56 ± 0.19 ^a^	1123.47 ± 386.26 ^a^
**Control**				
1 h	90.48 ± 8.25 ^a^ ^b^	0.24 ± 0.01 ^b^	4.23 ± 0.24 ^a^ ^b^	873.88 ± 81.38 ^a^
2 h	95.24 ± 8.25 ^a^	0.23 ± 0.01 ^a^ ^b^	4.33 ± 0.12 ^a^ ^b^	1225.99 ± 297.13 ^a^
3 h	100 ± 0 ^a^	0.22 ± 0.00 ^a^	4.52 ± 0.09 ^a^	1223.33 ± 85.31 ^a^
4 h	90.48 ± 16.50 ^a^ ^b^	0.23 ± 0.01 ^a^ ^b^	4.38 ± 0.17 ^a^ ^b^	1306.05 ± 652.12 ^a^
5 h	76.19 ± 8.25 ^b^	0.23 ± 0.00 ^a^ ^b^	4.28 ± 0.09 ^b^	892.99 ± 256.88 ^a^

Within columns, values with different and same superscript letters represent differences that are significantly and not significantly different, respectively (p < 0.05).

The germination rate values across all treatment clustered around 0.21–0.24 d ^−1^, consistently matching the control setup. There was no consistent pattern observed with respect to steeping time ( Table 10).

For germination time, the control value was recorded between 4.23-4.52 d, while generally the germination time maintained a stable trend for all treatment groups ( Table 10).

Vigor index with the highest value was observed for *P. aeruginosa* PV936736 (1386.60) at 1 h which is closely related to the control value (1306.05). *E. hormaechei* showed an upward trend from 780.48 at 1 h to 1247.62 at 5 h steeping period. *P. aeruginosa* PV936739 displayed a sharp decline in vigor (133.88) at 3 h ( Table 10).

***Sorghum***

For the sorghum seeds, with respect to germination, most treatments germination (often >70%) exceeding the control value of 71.43%. *P. aeruginosa* (PV936735) and *E. hormaechei* (PV936738) showed consistently high germination (85.71–90.48%) across all steeping periods. *P. aeruginosa* (PV936736) ranged widely with a low germination at 1 h (57.14%) to an increased germination (100%) at 2 h, with the 1 h and 4 h treatments significantly lower than the other steeping times ( Table 11).

**Table 11.** Effect of steeping time on phytotoxicity of the sorghum seeds in presence of the test bacterial strains.

Steeping time	% germination	Germination rate	Germination Time	Vigor index
***Pseudomonas aeruginosa* (PV936735)**				
1 h	85.71 ± 14.29 ^a^	0.23 ± 0.01 ^a^	4.30 ± 0.18 ^a^	481.56 ± 43.31 ^b^
2 h	90.48 ± 16.50 ^a^	0.24 ± 0.00 ^a^	4.15 ± 0.07 ^a^	894.15 ± 181.36 ^a^
3 h	85.71 ± 14.29 ^a^	0.23 ± 0.01 ^a^	4.30 ± 0.13 ^a^	589.59 ± 157.41 ^a^ ^b^
4 h	85.71 ± 14.29 ^a^	0.23 ± 0.00 ^a^	4.32 ± 0.03 ^a^	691.77 ± 288.53 ^a^ ^b^
5 h	90.48 ± 8.25 ^a^	0.25 ± 0.00 ^b^	4.07 ± 0.07 ^b^	710.14 ± 59.23 ^a^ ^b^
***Pseudomonas aeruginosa* (PV936736)**				
1 h	57.14 ± 14.29 ^c^	0.24 ± 0.01 ^a^	4.10 ± 0.11 ^a^	227.42 ± 115.75 ^c^
2 h	100 ± 0.00 ^a^	0.24 ± 0.01 ^a^	4.13 ± 0.11 ^a^	956.67 ± 68.70 ^a^
3 h	95.24 ± 8.25 ^a^ ^b^	0.24 ± 0.01 ^a^	4.15 ± 0.13 ^a^	848.84 ± 190.14 ^a^ ^b^
4 h	66.67 ± 8.25 ^b^	0.24 ± 0.00 ^a^	4.20 ± 0.03 ^a^	375.31 ± 112.45 ^b^
5 h	95.24 ± 8.25 ^a^ ^b^	0.24 ± 0.00 ^a^	4.17 ± 0.07 ^a^	669.93 ± 62.89 ^b^
***Pseudomonas fluorescens* (PV936737)**				
1 h	76.19 ± 21.82 ^b^	0.24 ± 0.01 ^a^	4.23 ± 0.13 ^a^ ^b^	478.10 ± 325.24 ^a^
2 h	95.24 ± 8.25 ^a^	0.23 ± 0.00 ^b^	4.26 ± 0.03 ^a^	708.64 ± 136.10 ^a^
3 h	90.48 ± 8.25 ^a^	0.24 ± 0.00 ^a^	4.18 ± 0.03 ^a^ ^b^	737.07 ± 181.91 ^a^
4 h	76.19 ± 8.25 ^b^	0.24 ± 0.00 ^a^	4.13 ± 0.07 ^b^	519.86 ± 191.72 ^a^
5 h	80.95 ± 8.25 ^b^	0.23 ± 0.00 ^b^	4.30 ± 0.02 ^a^	419.52 ± 81.50 ^a^
***Enterobacter hormaechei* (PV936738)**				
1 h	85.71 ± 14.29 ^a^	0.24 ± 0.01 ^b^	4.12 ± 0.11 ^b^	455.31 ± 160.67 ^c^
2 h	95.24 ± 8.25 ^a^	0.23 ± 0.00 ^c^	4.32 ± 0.08 ^a^	747.48 ± 244.44 ^a^
3 h	95.24 ± 8.25 ^a^	0.25 ± 0.01 ^a^	4.08 ± 0.09 ^b^	829.05 ± 111.94 ^a^
4 h	95.24 ± 6.25 ^a^	0.25 ± 0.00 ^a^	4.08 ± 0.07 ^b^	759.66 ± 75.31 ^a^
5 h	90.48 ± 8.25 ^a^	0.23 ± 0.01 ^c^	4.27 ± 0.11 ^a^	533.81 ± 136.76 ^b^
***Pseudomonas aeruginosa* (PV936739)**				
1 h	76.19 ± 8.25 ^b^	0.24 ± 0.01 ^a^	4.14 ± 0.10 ^a^	573.13 ± 102.02 ^a^
2 h	95.24 ± 8.25 ^a^	0.24 ± 0.01 ^a^	4.21 ± 0.14 ^a^	754.29 ± 49.14 ^a^
3 h	100 ± 0.00 ^a^	0.24 ± 0.01 ^a^	4.11 ± 0.10 ^a^	895.71 ± 85.10 ^a^
4 h	80.95 ± 16.50 ^b^	0.25 ± 0.01 ^a^	4.06 ± 0.11 ^a^	584.69 ± 326.32 ^a^
5 h	95.24 ± 8.25 ^a^	0.24 ± 0.01 ^a^	4.16 ± 0.14 ^a^	588.98 ± 185.33 ^a^
**Control**				
1 h	71.42 ± 24.74 ^a^ ^b^	0.25 ± 0.00 ^a^	4.07 ± 0.06 ^b^	437.76 ± 268.75 ^a^ ^b^
2 h	76.19 ± 8.25 ^a^ ^b^	0.23 ± 0.00 ^c^	4.31 ± 0.06 ^a^	527.55 ± 207.88 ^a^ ^b^
3 h	85.71 ± 14.29 ^a^	0.24 ± 0.00 ^b^ ^c^	4.09 ± 0.06 ^b^	533.20 ± 122.25 ^a^ ^b^
4 h	57.14 ± 14.29 ^b^	0.25 ± 0.00 ^a^ ^b^	4.00 ± 0.06 ^b^	227.01 ± 77.29 ^b^
5 h	85.71 ± 0.00 ^a^	0.24 ± 0.00 ^b^ ^c^	4.12 ± 0.06 ^b^	586.12 ± 64.44 ^a^

Within columns, values with different and same superscript letters represent differences that are significantly and not significantly different, respectively (p < 0.05).

Germination rate remained steady across isolates, generally between 0.23–0.25, which aligns closely with the control (0.25), and showing modest influence of steeping duration. Germination time clustered between 4.06–4.32 d which is in close range with the control mean (4.07 d). *P. aeruginosa* (PV936735) showed a slight decline in the germination time at 5 h steeping period ( Table 11).

Remarkable phytotoxic effects were observed across all treatments at 1 h steeping time. Across all isolates, *E. hormaechei* showed the highest vigor index at 3 h (829.05) while the lowest vigor index was observed for *P. aeruginosa* (PV936736) at 1 h (227.42). For *P. aeruginosa* (PV936739) high vigor values which was consistently above the control (437.76) was observed indicating no phytotoxic effect across the steeping period ( Table 11).

***Cucumber***

Cucumber seeds had high germinability (≥85%) across all bacterial treatments and steeping durations, with *P. fluorescens* yielding the highest value at 1 h (1889.05). High germination percentages were observed for all treatments showing consistency with the control value (100%). *P. aeruginosa* (PV936736) constantly achieved 100% germination at all steeping times, while *P. fluorescens* (PV936737) showed a slight decline after 3 h steeping time ( Table 12).

**Table 12.** Effect of steeping time on phytotoxicity of the cucumber seeds in presence of the test bacterial strains.

Steeping time	% Germination	Germination rate	Germination time	Vigor index
***Pseudomonas aeruginosa* (PV936735)**				
1 h	95.24 ± 8.25 ^a^	0.24 ± 0.01 ^a^ ^b^	4.23 ± 0.13 ^b^	1124.69 ± 179.44 ^a^
2 h	95.24 ± 8.25 ^a^	0.23 ± 0.01 ^a^ ^b^	4.33 ± 0.14 ^b^	945.31 ± 230.85 ^a^
3 h	85.71 ± 14.29 ^a^	0.23 ± 0.01 ^a^ ^b^	4.38 ± 0.14 ^b^	909.18 ± 276.28 ^a^
4 h	85.71 ± 24.74 ^a^	0.23 ± 0.00 ^a^ ^b^	4.30 ± 0.05 ^b^	831.56 ± 397.72 ^a^
5 h	100 ± 0.00 ^a^	0.22 ± 0.00 ^b^	4.51 ± 0.07 ^a^	968.10 ± 121.44 ^a^
***Pseudomonas aeruginosa* (PV936736)**				
1 h	100 ± 0.00 ^a^	0.23 ± 0.01 ^a^	4.32 ± 0.10 ^a^	1135.24 ± 107.25 ^a^
2 h	100 ± 0.00 ^a^	0.23 ± 0.00 ^a^	4.39 ± 0.08 ^a^	1124.76 ± 87.61 ^a^
3 h	100 ± 0.00 ^a^	0.23 ± 0.00 ^a^	4.38 ± 0.05 ^a^	1196.19 ± 91.39 ^a^
4 h	100 ± 0.00 ^a^	0.23 ± 0.01 ^a^	4.45 ± 0.22 ^a^	1050.00 ± 84.50 ^a^
5 h	100 ± 0.00 ^a^	0.23 ± 0.01 ^a^	4.33 ± 0.13 ^a^	1157.62 ± 145.87 ^a^
***Pseudomonas fluorescens* (PV936737)**				
1 h	100 ± 0.00 ^a^	0.24 ± 0.01 ^a^	4.25 ± 0.11 ^a^	1889.05 ± 1199.10 ^a^
2 h	85.71 ± 14.29 ^a^ ^b^	0.23 ± 0.00 ^a^ ^b^	4.35 ± 0.00 ^a^ ^b^	858.64 ± 198.38 ^b^
3 h	100 ± 0.00 ^a^	0.21 ± 0.01 ^c^	4.66 ± 0.13 ^c^	1004.29 ± 164.15 ^b^
4 h	85.71 ± 14.29 ^a^ ^b^	0.22 ± 0.01 ^b^ ^c^	4.50 ± 0.15 ^b^ ^c^	724.15 ± 367.39 ^b^
5 h	80.95 ± 8.25 ^b^	0.23 ± 0.01 ^a^ ^b^	4.46 ± 0.20 ^b^ ^c^	720.00 ± 246.92 ^b^
***Enterobacter hormaechei* (PV936738)**				
1 h	85.71 ± 14.29 ^a^	0.23 ± 0.01 ^a^ ^b^	4.28 ± 0.10 ^a^ ^b^	949.52 ± 485.64 ^a^
2 h	100 ± 0.00 ^a^	0.23 ± 0.01 ^a^ ^b^	4.34 ± 0.15 ^a^ ^b^	1081.43 ± 109.33 ^a^
3 h	85.71 ± 14.29 ^a^	0.22 ± 0.01 ^a^ ^b^	4.54 ± 0.23 ^a^ ^b^	812.79 ± 349.32 ^a^
4 h	100 ± 0.00 ^a^	0.23 ± 0.00 ^a^ ^b^	4.32 ± 0.05 ^a^ ^b^	1246.67 ± 11.90 ^a^
5 h	95.24 ± 8.25 ^a^	0.20 ± 0.01 ^b^	4.55 ± 0.15 ^b^	987.21 ± 254.16 ^a^
***Pseudomonas aeruginosa* (PV936739)**				
1 h	90.48 ± 8.25 ^a^	0.22 ± 0.00 ^a^	4.51 ± 0.05 ^a^	921.50 ± 151.43 ^a^
2 h	90.48 ± 8.25 ^a^	0.23 ± 0.02 ^a^	4.42 ± 0.30 ^a^	917.69 ± 190.70 ^a^
3 h	90.48 ± 16.50 ^a^	0.22 ± 0.01 ^a^	4.45 ± 0.24 ^a^	938.64 ± 251.25 ^a^
4 h	95.24 ± 8.25 ^a^	0.22 ± 0.00 ^a^	4.50 ± 0.06 ^a^	999.80 ± 208.77 ^a^
5 h	85.71 ± 24.74 ^a^	0.23 ± 0.01 ^a^	4.43 ± 0.18 ^a^	770.48 ± 469.86 ^a^
**Control**				
1 h	100 ± 0.00 ^a^	0.23 ± 0.01 ^a^	4.32 ± 0.16 ^b^	948.10 ± 179.30 ^a^
2 h	100 ± 0.00 ^a^	0.23 ± 0.00 ^a^	4.29 ± 0.04 ^b^	1293.81 ± 61.02 ^a^
3 h	100 ± 0.00 ^a^	0.23 ± 0.00 ^a^	4.42 ± 0.05 ^b^	1153.33 ± 74.84 ^a^
4 h	80.95 ± 32.99 ^a^	0.24 ± 0.00 ^b^	4.09 ± 0.07 ^a^	825.24 ± 714.72 ^a^
5 h	76.19 ± 16.50 ^a^	0.23 ± 0.00 ^a^	4.27 ± 0.07 ^b^	682.31 ± 459.53 ^a^

Within columns, values with different and same superscript letters represent differences that are significantly and not significantly different, respectively (p < 0.05).

Germination rate clustered around 0.20–0.24 similar to the control value (0.23). *P. aeruginosa* (PV936736) showed a constant germination rate (0.23) indicating no influence of steeping period, *E. hormaechei* (PV936738) showed the lowest germination rate at 5 h (0.20). For germination time the recorded value for the control mean was 4.32 d, while shorter steeping period (1–2 h) reduced germination time to between 4.2–4.3 d (notably *P. fluorescens* (PV936737) and *E. hormaechei* (PV936738), longer steeping period (3-5 h) prolonged germination time. *P. fluorescens* (PV936737) displayed the longest germination time (4.66 d at 3 h). Among the strains, *P. fluorescens* (PV936737) produced the highest early vigor (1889 at 1 h) but dropped sharply to 720 at 5 h steeping time, indicating phytotoxic effect at prolonged steeping. *P. aeruginosa* PV936736 consistently showed strong vigor index (>1000) across all steeping periods ( Table 12).

**3.3.3 Effect of inoculum concentration**

***Cowpea***

For cowpea, high inoculum concentrations (100:0) showed reduction in germination and vigor. At medium dilutions (80:20, 60:40, 20:80) improved germination% and vigor index were observed. However, germination rate and time are not generally affected, showing no significant changes ( Table 13).

**Table 13.** Effect of inoculum concentration on phytotoxicity of the cowpea seeds in presence of the test bacterial strains.

Inoculum dilution	% germination	Germination rate	Germination time	Vigor index
***Pseudomonas aeruginosa* (PV936735)**				
100:0	61.90 ± 16.50 ^a^	0.23 ± 0.01 ^a^	4.36 ± 0.26 ^a^	458.84 ± 212.34 ^b^
80:20	95.24 ± 8.25 ^b^	0.23 ± 0.01 ^a^	4.36 ± 0.30 ^a^	1556.12 ± 530.10 ^a^
60:40	75.40 ± 15.85 ^a^ ^b^	0.24 ± 0.00 ^a^	4.23 ± 0.05 ^a^	1079.52 ± 321.70 ^a^ ^b^
40:60	90.48 ± 8.25 ^b^	0.23 ± 0.00 ^a^	4.43 ± 0.04 ^a^	1556.67 ± 226.65 ^a^
20:80	95.24 ± 8.25 ^b^	0.22 ± 0.01 ^a^	4.47 ± 0.17 ^a^	1448.44 ± 277.76 ^a^
10:100	71.43 ± 14.29 ^a^ ^b^	0.23 ± 0.01 ^a^	4.31 ± 0.19 ^a^	671.84 ± 486.04 ^b^
Control	100 ± 0.00 ^b^	0.22 ± 0.01 ^a^	4.45 ± 0.18 ^a^	1611.90 ± 158.06 ^a^
***Pseudomonas aeruginosa* (PV936736)**				
100:0	85.71 ± 14.29 ^a^ ^b^	0.23 ± 0.01 ^a^	4.42 ± 0.10 ^a^	1147.96 ± 485.24 ^b^
80:20	90.48 ± 8.25 ^a^ ^b^	0.23 ± 0.00 ^a^	4.41 ± 0.04 ^a^	1606.19 ± 464.13 ^a^
60:40	95.24 ± 8.25 ^a^	0.22 ± 0.01 ^a^	4.47 ± 0.12 ^a^	1407.35 ± 443.90 ^a^ ^b^
40:60	66.67 ± 16.50 ^c^	0.23 ± 0.01 ^a^	4.31 ± 0.15 ^a^	617.14 ± 130.76 ^c^
20:80	95.24 ± 8.25 ^a^	0.24 ± 0.00 ^a^	4.23 ± 0.09 ^a^	1678.10 ± 384.23 ^a^
10:100	85.71 ± 14.29 ^a^ ^b^	0.23 ± 0.01 ^a^	4.44 ± 0.21 ^a^	1307.76 ± 274.10 ^a^ ^b^
Control	85.71 ± 24.74 ^a^ ^b^	0.22 ± 0.01 ^a^	4.47 ± 0.11 ^a^	1267.94 ± 605.03 ^b^
***Pseudomonas fluorescens* (PV936737)**				
100:0	85.71 ± 0.00 ^a^ ^b^	0.23 ± 0.01 ^a^	4.45 ± 0.13 ^a^ ^b^	1273.13 ± 79.12 ^a^ ^b^
80:20	71.43 ± 14.29 ^b^	0.22 ± 0.00 ^a^ ^b^	4.64 ± 0.07 ^a^	953.95 ± 406.26 ^b^
60:40	100 ± 0.00 ^a^	0.22 ± 0.01 ^a^ ^b^	4.64 ± 0.15 ^a^	1304.29 ± 323.31 ^a^ ^b^
40:60	85.71 ± 14.29 ^a^ ^b^	0.23 ± 0.01 ^a^	4.30 ± 0.15 ^b^	1193.16 ± 439.05 ^a^ ^b^
20:80	80.95 ± 8.25 ^a^ ^b^	0.23 ± 0.01 ^a^	4.41 ± 0.23 ^a^ ^b^	1193.71 ± 368.39 ^a^ ^b^
10:100	100 ± 0.00 ^a^	0.21 ± 0.01 ^b^	4.74 ± 0.14 ^a^	1579.05 ± 151.96 ^a^
Control	95.24 ± 8.25 ^a^	0.23 ± 0.01 ^a^	4.31 ± 0.18 ^b^	1567.42 ± 203.87 ^a^
***Enterobacter hormaechei* (PV936738)**				
100:0	85.71 ± 14.29 ^a^	0.22 ± 0.00 ^a^	4.49 ± 0.08 ^a^	1356.53 ± 468.99 ^a^
80:20	80.95 ± 8.25 ^a^	0.23 ± 0.00 ^a^	4.31 ± 0.09 ^a^	1125.71 ± 422.69 ^a^
60:40	90.48 ± 8.25 ^a^	0.23 ± 0.00 ^a^	4.39 ± 0.06 ^a^	1415.17 ± 301.01 ^a^
40:60	90.48 ± 16.50 ^a^	0.23 ± 0.00 ^a^	4.42 ± 0.09 ^a^	1446.05 ± 521.78 ^a^
20:80	80.95 ± 32.99 ^a^	0.23 ± 0.01 ^a^	4.27 ± 0.24 ^a^	1298.33 ± 888.33 ^a^
10:100	95.24 ± 8.25 ^a^	0.23 ± 0.01 ^a^	4.37 ± 0.17 ^a^	1525.37 ± 289.46 ^a^
Control	90.48 ± 8.25 ^a^	0.23 ± 0.01 ^a^	4.44 ± 0.17 ^a^	1186.12 ± 217.15 ^a^
***Pseudomonas aeruginosa* (PV936739)**				
100:0	80.16 ± 7.65 ^b^	0.20 ± 0.01 ^b^	4.89 ± 0.13 ^a^	908.72 ± 88.92 ^b^
80:20	95.24 ± 8.25 ^a^	0.24 ± 0.01 ^a^	4.24 ± 0.11 ^b^	1122.31 ± 718.88 ^a^ ^b^
60:40	95.24 ± 8.25 ^a^	0.24 ± 0.01 ^a^	4.16 ± 0.15 ^b^	1777.89 ± 415.68 ^a^
40:60	95.24 ± 8.25 ^a^	0.23 ± 0.01 ^a^	4.31 ± 0.20 ^b^	1532.38 ± 302.93 ^a^ ^b^
20:80	80.95 ± 8.25 ^b^	0.23 ± 0.00 ^a^	4.27 ± 0.08 ^b^	1352.19 ± 300.08 ^a^ ^b^
10:100	100 ± 0.00 ^a^	0.23 ± 0.01 ^a^	4.27 ± 0.09 ^b^	1651.35 ± 287.89 ^a^
Control	95.24 ± 8.25 ^a^	0.24 ± 0.01 ^a^	4.23 ± 0.10 ^b^	1402.79 ± 468.03 ^a^ ^b^

Within columns, values with different and same superscript letters represent differences that are significantly and not significantly different, respectively (p < 0.05). Dilution ratios connote inoculum: water ratio.

In treatment with the *P. aeruginosa* (PV936735), lowest% germination was recorded at 100% inoculum (61.9%), which improved significantly to 95.24% at 80:20 and 20:80 dilutions and 100% for the control. Germination rate and time remain stable across dilutions. As was observed for germination, vigor index showed lowest value at 100% inoculum and was significantly higher and comparable to control at 80:20, 40:60, and 20:80 dilutions, suggesting reduced phytotoxicity at these dilutions ( Table 13).

For *P. aeruginosa* (PV936736), fluctuation in germination was observed across the respective inoculums. Highest values were however recorded at 60:40 and 20:80 dilutions (95%), but dropped significantly at 40:60 (66.67%). In the case of vigor index, significantly higher values were recorded at 20:80 dilution (1678) and 80:20 (1606), which dropped 40:60 dilution ( Table 13).

In presence of the *P. fluorescens* (PV936737),% germination showed relatively low values across high dilutions, reaching 100% at 60:40 and 10:100 ratios. In addition, relatively longer germination time was observed at higher dilutions (80:20 and 60:40). For vigor index, a decrease was observed at 80:20 but was higher at 10:100 and close to control at several dilutions ( Table 13).

Seeds treated with the *E. hormaechei* (PV936738) showed stable% germination of around 80–95% at the different dilutions with germination rate and time showing minimal variation. In addition, vigor index showed no significant variation across the different dilutions ( Table 13).

In the case of the P. aeruginosa (PV936739) treated seeds,% germination showed increase from 80% at 100% inoculum to above 95% at some of the other dilutions, except a slight dip at 20:80. Also, germination rate showed lowest value at 100% inoculum (0.20) but showed improvement at reduced dilutions, with observed significant at 60:40 dilution (1777) ( Table 13).

***Maize***

Overall, the maize seeds showed slight sensitivity to inoculum concentration, with high inoculum occasionally reducing seed performance. Only slight sensitivity to inoculum concentration was observed for % germination while vigor index was negatively affected at certain dilutions, such as 80:20. Germination rate and time were however observed to be stable across the different dilutions ( Table 14).

**Table 14.** Effect of inoculum concentration on phytotoxicity of the maize seeds in presence of the test bacterial strains.

Inoculum dilution	% germination	Germination rate	Germination time	Vigor index
***Pseudomonas aeruginosa* (PV936735)**				
100:0	85.71 ± 14.29 ^a^ ^b^	0.21 ± 0.00 ^a^	4.82 ± 0.04 ^a^	620.64 ± 108.61 ^a^
80:20	66.67 ± 8.25 ^c^	0.21 ± 0.01 ^a^	4.86 ± 0.16 ^a^	425.31 ± 91.99 ^a^
60:40	69.84 ± 14.55 ^b^ ^c^	0.21 ± 0.01 ^a^	4.81 ± 0.22 ^a^	492.94 ± 248.58 ^a^
40:60	81.35 ± 5.63 ^a^ ^b^	0.20 ± 0.01 ^a^	4.89 ± 0.22 ^a^	594.34 ± 111.12 ^a^
20:80	88.57 ± 10.30 ^a^	0.20 ± 0.01 ^a^	5.06 ± 0.24 ^a^	723.57 ± 408.88 ^a^
10:100	76.59 ± 6.11 ^a^ ^b^	0.21 ± 0.01 ^a^	4.72 ± 0.18 ^a^	545.68 ± 109.38 ^a^
0:100	94.44 ± 9.62 ^a^	0.22 ± 0.02 ^a^	4.51 ± 0.42 ^a^	687.78 ± 303.57 ^a^
***Pseudomonas aeruginosa* (PV936736)**				
100:0	84.92 ± 1.37 ^a^	0.20 ± 0.00 ^a^	4.96 ± 0.11 ^a^	536.40 ± 207.29 ^a^
80:20	74.60 ± 9.91 ^a^	0.21 ± 0.00 ^a^	4.79 ± 0.10 ^a^	519.13 ± 185.85 ^a^
60:40	69.05 ± 17.98 ^a^	0.20 ± 0.00 ^a^	4.89 ± 0.10 ^a^	424.42 ± 339.37 ^a^
40:60	80.95 ± 16.50 ^a^	0.20 ± 0.00 ^a^	4.95 ± 0.09 ^a^	601.09 ± 382.18 ^a^
20:80	75.40 ± 15.85 ^a^	0.20 ± 0.00 ^a^	4.89 ± 0.07 ^a^	501.29 ± 191.91 ^a^
10:100	65.71 ± 30.90 ^a^	0.20 ± 0.01 ^a^	5.06 ± 0.33 ^a^	398.11 ± 416.10 ^a^
0:100	90.48 ± 8.25 ^a^	0.20 ± 0.01 ^a^	4.88 ± 0.13 ^a^	785.03 ± 178.41 ^a^
***Pseudomonas fluorescens* (PV936737)**				
100:0	76.60 ± 9.91 ^a^	0.22 ± 0.01 ^a^	4.59 ± 0.25 ^b^	537.07 ± 259.08 ^a^
80:20	64.29 ± 12.37 ^a^	0.20 ± 0.01 ^b^	4.98 ± 0.21 ^a^	360.64 ± 158.27 ^a^
60:40	71.43 ± 24.74 ^a^	0.19 ± 0.01 ^c^	5.27 ± 0.35 ^c^	425.10 ± 295.46 ^a^
40:60	80.95 ± 16.50 ^a^	0.21 ± 0.00 ^a^ ^b^	4.88 ± 0.04 ^a^	663.54 ± 275.41 ^a^
20:80	89.68 ± 9.01 ^a^	0.21 ± 0.01 ^a^ ^b^	4.86 ± 0.15 ^a^	765.09 ± 24.64 ^a^
10:100	66.35 ± 28.73 ^a^	0.20 ± 0.00 ^b^	4.89 ± 0.11 ^a^	512.39 ± 338.52 ^a^
0:100	90.48 ± 8.25 ^a^	0.21 ± 0.00 ^a^ ^b^	4.83 ± 0.03 ^a^	725.37 ± 98.67 ^a^
***Enterobacter hormaechei* (PV936738)**				
100:0	75.40 ± 29.39 ^a^	0.21 ± 0.00 ^a^	4.68 ± 0.07 ^a^ ^b^	472.24 ± 239.86 ^a^
80:20	80.95 ± 16.50 ^a^	0.20 ± 0.01 ^a^ ^b^	4.97 ± 0.28 ^a^	596.12 ± 337.34 ^a^
60:40	76.19 ± 16.50 ^a^	0.19 ± 0.01 ^b^	5.15 ± 0.27 ^b^	436.05 ± 278.86 ^a^
40:60	69.84 ± 14.55 ^a^	0.21 ± 0.01 ^a^	4.80 ± 0.14 ^a^ ^b^	508.91 ± 206.53 ^a^
20:80	74.60 ± 9.91 ^a^	0.21 ± 0.00 ^a^ ^b^	4.87 ± 0.12 ^a^ ^b^	441.44 ± 81.52 ^a^
10:100	95.24 ± 8.25 ^a^	0.21 ± 0.00 ^a^ ^b^	4.84 ± 0.09 ^a^ ^b^	908.84 ± 296.50 ^a^
0:100	71.43 ± 37.80 ^a^	0.20 ± 0.00 ^a^ ^b^	4.95 ± 0.67 ^a^	474.43 ± 361.38 ^a^
***Pseudomonas aeruginosa* (PV936739)**				
100:0	44.44 ± 11.98 ^c^	0.21 ± 0.01 ^a^ ^b^	4.66 ± 0.14 ^a^ ^b^	222.55 ± 146.19 ^c^
80:20	80.16 ± 7.65 ^a^	0.21 ± 0.01 ^a^ ^b^	4.78 ± 0.15 ^a^ ^b^	460.27 ± 70.28 ^b^
60:40	76.19 ± 8.25 ^a^ ^b^	0.21 ± 0.01 ^a^ ^b^	4.83 ± 0.21 ^a^ ^b^	531.90 ± 214.87 ^b^
40:60	70.24 ± 18.33 ^b^	0.21 ± 0.00 ^a^ ^b^	4.75 ± 0.00 ^a^ ^b^	437.06 ± 300.31 ^b^
20:80	90.48 ± 16.50 ^a^	0.21 ± 0.01 ^a^ ^b^	4.87 ± 0.15 ^a^ ^b^	707.76 ± 279.89 ^a^ ^b^
10:100	88.57 ± 10.30 ^a^	0.21 ± 0.01 ^a^ ^b^	4.87 ± 0.23 ^a^ ^b^	682.70 ± 155.42 ^a^ ^b^
0:100	80.95 ± 8.25 ^a^ ^b^	0.20 ± 0.00 ^b^	4.97 ± 0.10 ^a^	892.04 ± 433.93 ^a^

Within columns, values with different and same superscript letters represent differences that are significantly and not significantly different, respectively (p < 0.05). Dilution ratios connote inoculum: water ratio.

In presence of the *P. aeruginosa* (PV936735), germination % showed wide variation but dropped significantly at 80:20 (66.67%) and 60:40 (69.84%) dilutions but was higher at 100:0 (85.71%) and 20:80 (88.57%). Germination rate and time were mostly stable across the different dilutions while reduction in vigor was observed at 80:20 dilution ( Table 14).

For the *P. aeruginosa* (PV936736), % germination was relatively high and stable across dilutions, although reduction was observed at 10:100. Germination rate and time however remained constant while variation in vigor was observed, although showed no consistent trend with inoculum concentrations ( Table 14).

In addition, % germination showed variation and slight decrease at some dilutions (80:20, 60:40) in presence of *P. fluorescens* (PV936737). Germination rate however showed minute reduction 60:40 dilution while germination time increased significantly at 60:40 dilution (5.27 d). In the case of vigor index, no consistent pattern of increase or decrease was observed ( Table 14).

When treated with the *E. hormaechei* (PV936738), variation in % germination was observed across the respective dilutions, ranging roughly 65–95% while germination rate showed slight decrease at 60:40. However, vigor index generally revealed no significant differences across the respective dilutions ( Table 14).

For the *P. aeruginosa* (PV936739) treated seeds, % germination was lowest at 100:0 (44.44%) and showed increases at dilutions (around 70–90%). Similarly, lowest vigor index was recorded lowest at 100% inoculum but germination rate and time did not show any variation ( Table 14).

***Soybean***

The general observation for the soybean seeds showed tolerance to different inoculum concentrations, with no significant negative impact on germinability parameters ( Table 15).

**Table 15.** Effect of inoculum concentration on phytotoxicity of the soybean seeds in presence of the test bacterial strains.

Inoculum dilution	% germination	Germination rate	Germination time	Vigor index
***Pseudomonas aeruginosa* (PV936735)**				
100:0	80.95 ± 8.25 ^a^	0.22 ± 0.01 ^a^ ^b^	4.57 ± 0.12 ^a^ ^b^	876.39 ± 322.74 ^a^
80:20	85.71 ± 0.00 ^a^	0.22 ± 0.00 ^a^ ^b^	4.54 ± 0.09 ^a^ ^b^	1108.16 ± 216.33 ^a^
60:40	76.19 ± 16.50 ^a^	0.23 ± 0.00 ^a^	4.37 ± 0.01 ^b^	1001.96 ± 205.39 ^a^
40:60	71.43 ± 14.29 ^a^	0.22 ± 0.01 ^a^ ^b^	4.62 ± 0.14 ^a^ ^b^	806.26 ± 420.03 ^a^
20:80	80.95 ± 8.25 ^a^	0.22 ± 0.01 ^a^ ^b^	4.54 ± 0.15 ^a^ ^b^	999.12 ± 263.45 ^a^
10:100	76.19 ± 29.74 ^a^	0.22 ± 0.01 ^a^ ^b^	4.49 ± 0.17 ^a^ ^b^	1106.87 ± 854.42 ^a^
0:100	85.71 ± 14.29 ^a^	0.23 ± 0.01 ^a^	4.37 ± 0.13 ^b^	1181.09 ± 454.60 ^a^
***Pseudomonas aeruginosa* (PV936736)**				
100:0	90.48 ± 8.25 ^a^	0.21 ± 0.01 ^a^	4.71 ± 0.27 ^a^	933.88 ± 200.79 ^a^
80:20	80.95 ± 16.50 ^a^	0.22 ± 0.01 ^a^	4.54 ± 0.29 ^a^	944.69 ± 193.22 ^a^
60:40	71.43 ± 14.29 ^a^	0.23 ± 0.00 ^a^	4.42 ± 0.07 ^a^	893.58 ± 217.76 ^a^
40:60	90.48 ± 8.25 ^a^	0.22 ± 0.00 ^a^	4.49 ± 0.06 ^a^	1336.16 ± 520.31 ^a^
20:80	85.71 ± 0.00 ^a^	0.22 ± 0.00 ^a^	4.57 ± 0.06 ^a^	970.61 ± 191.87 ^a^
10:100	85.71 ± 14.29 ^a^	0.22 ± 0.01 ^a^	4.52 ± 0.22 ^a^	1118.97 ± 336.89 ^a^
0:100	85.71 ± 14.29 ^a^	0.22 ± 0.01 ^a^	4.57 ± 0.22 ^a^	1213.95 ± 561.64 ^a^
***Pseudomonas fluorescens* (PV936737)**				
100:0	90.48 ± 8.25 ^a^	0.22 ± 0.00 ^a^	4.48 ± 0.05 ^a^	1039.31 ± 385.30 ^a^
80:20	85.71 ± 14.29 ^a^	0.22 ± 0.00 ^a^	4.54 ± 0.04 ^a^	1210.61 ± 612.68 ^a^
60:40	76.19 ± 8.25 ^a^	0.23 ± 0.00 ^a^	4.40 ± 0.06 ^a^	902.79 ± 297.05 ^a^
40:60	95.24 ± 8.25 ^a^	0.23 ± 0.00 ^a^	4.44 ± 0.07 ^a^	1372.38 ± 267.05 ^a^
20:80	85.71 ± 14.29 ^a^	0.22 ± 0.00 ^a^	4.49 ± 0.09 ^a^	1224.78 ± 330.54 ^a^
10:100	76.19 ± 29.74 ^a^	0.23 ± 0.01 ^a^	4.38 ± 0.17 ^a^	1105.44 ± 647.39 ^a^
0:100	71.43 ± 14.29 ^a^	0.22 ± 0.01 ^a^	4.48 ± 0.19 ^a^	889.05 ± 288.11 ^a^
***Enterobacter hormaechei* (PV936738)**				
100:0	80.95 ± 8.25 ^a^	0.22 ± 0.02 ^a^	4.55 ± 0.35 ^a^	886.80 ± 264.16 ^b^
80:20	95.24 ± 8.25 ^a^	0.23 ± 0.00 ^a^	4.42 ± 0.09 ^a^	1466.26 ± 381.80 ^a^
60:40	80.95 ± 8.25 ^a^	0.22 ± 0.01 ^a^	4.52 ± 0.22 ^a^	906.19 ± 257.75 ^a^ ^b^
40:60	90.48 ± 8.25 ^a^	0.23 ± 0.01 ^a^	4.42 ± 0.16 ^a^	1032.99 ± 269.84 ^a^ ^b^
20:80	85.71 ± 14.29 ^a^	0.22 ± 0.01 ^a^	4.46 ± 0.20 ^a^	1160.48 ± 230.03 ^a^ ^b^
10:100	95.24 ± 8.25 ^a^	0.22 ± 0.01 ^a^	4.52 ± 0.11 ^a^	1418.98 ± 254.31 ^a^ ^b^
0:100	90.48 ± 8.25 ^a^	0.22 ± 0.01 ^a^	4.61 ± 0.34 ^a^	1145.10 ± 505.11 ^a^ ^b^
***Pseudomonas aeruginosa* (PV936739)**				
100:0	76.19 ± 8.25 ^a^	0.22 ± 0.00 ^a^ ^b^	4.60 ± 0.08 ^a^ ^b^	648.37 ± 183.72 ^a^
80:20	80.95 ± 8.25 ^a^	0.22 ± 0.01 ^a^ ^b^	4.56 ± 0.14 ^a^ ^b^	775.51 ± 225.84 ^a^
60:40	76.19 ± 21.82 ^a^	0.22 ± 0.01 ^a^ ^b^	4.51 ± 0.19 ^a^ ^b^	871.76 ± 543.83 ^a^
40:60	76.19 ± 29.74 ^a^	0.22 ± 0.00 ^a^ ^b^	4.57 ± 0.07 ^a^ ^b^	909.93 ± 587.55 ^a^
20:80	71.43 ± 37.80 ^a^	0.23 ± 0.01 ^b^	4.38 ± 0.17 ^b^	768.37 ± 602.10 ^a^
10:100	76.19 ± 16.50 ^a^	0.21 ± 0.02 ^a^	4.78 ± 0.46 ^a^	691.56 ± 573.67 ^a^
0:100	66.67 ± 8.25 ^a^	0.22 ± 0.00 ^a^ ^b^	4.45 ± 0.08 ^a^ ^b^	1327.76 ± 1011.45 ^a^

Within columns, values with different and same superscript letters represent differences that are significantly and not significantly different, respectively (p < 0.05). Dilution ratios connote inoculum: water ratio.

The *P. aeruginosa* (PV936735) treated seeds showed stable % germination (70–85%) with no significant decrease with inoculum concentration. Germination rate and time consistently revealed slight decrease in vigor index at 40:60 dilution ( Table 15).

For *P. aeruginosa* (PV936736), % germination ranged from 71 to 90% and was stable across dilutions. In the case vigor index, highest value was recorded at 40:60 (1336) and showed close value to the control at other dilutions ( Table 15).

In the presence of *P. fluorescens* (PV936737), % germination showed stability across all dilutions while highest vigor index was recorded at 40:60 dilution. Vigor was also high at other dilutions with no clear phytotoxic trend ( Table 15).

The *E. hormaechei* (PV936738) treated seeds stable but high % germination % (80–95%) at the respective dilutions. In the case of vigor index, no clear pattern of increases or decreases was observed with dilution ( Table 15).

Also, seeds treated with the *P. aeruginosa* (PV936739) revealed consistent % germination at the different inoculums (65–85%) while vigor index revealed variability, although with no significant reduction at any dilution ( Table 15).

***Sorghum***

Generally, the sorghum seeds showed some sensitivity to inoculum concentration, especially with changes in vigor index, where reduction in values were recorded with intermediate dilutions, despite high % germination ( Table 16).

**Table 16.** Effect of inoculum concentration on phytotoxicity of the sorghum seeds in presence of the test bacterial strains.

Inoculum dilution	% germination	Germination rate	Germination time	Vigor Index
***Pseudomonas aeruginosa* (PV936735)**				
100:0	100 ± 0.00 ^a^	0.23 ± 0.01 ^a^	4.45 ± 0.25 ^a^	588.06 ± 210.51 ^a^
80:20	84.13 ± 1.37 ^a^ ^b^	0.24 ± 0.00 ^a^	4.22 ± 0.08 ^a^	528.82 ± 58.63 ^a^
60:40	72.22 ± 34.69 ^b^	0.24 ± 0.02 ^a^	4.22 ± 0.37 ^a^	418.98 ± 334.91 ^a^
40:60	80.16 ± 7.65 ^a^ ^b^	0.22 ± 0.01 ^a^	4.52 ± 0.22 ^a^	336.53 ± 71.41 ^a^
20:80	69.05 ± 17.98 ^b^	0.23 ± 0.01 ^a^	4.41 ± 0.10 ^a^	422.10 ± 237.43 ^a^
10:100	94.44 ± 9.62 ^a^ ^b^	0.23 ± 0.00 ^a^	4.30 ± 0.07 ^a^	578.54 ± 150.75 ^a^
0:100	95.24 ± 8.25 ^a^ ^b^	0.22 ± 0.00 ^a^	4.50 ± 0.07 ^a^	563.72 ± 149.35 ^a^
***Pseudomonas aeruginosa* (PV936736)**				
100:0	90.48 ± 16.50 ^a^	0.22 ± 0.01 ^a^	4.61 ± 0.22 ^a^	428.84 ± 161.00 ^a^
80:20	80.95 ± 32.99 ^a^	0.23 ± 0.00 ^a^	4.37 ± 0.07 ^a^	454.83 ± 244.90 ^a^
60:40	90.48 ± 16.50 ^a^	0.23 ± 0.01 ^a^	4.44 ± 0.14 ^a^	573.88 ± 247.45 ^a^
40:60	66.67 ± 8.25 ^a^	0.22 ± 0.00 ^a^	4.47 ± 0.09 ^a^	296.39 ± 105.29 ^a^
20:80	85.71 ± 14.29 ^a^	0.23 ± 0.00 ^a^	4.42 ± 0.04 ^a^	487.89 ± 158.03 ^a^
10:100	85.71 ± 14.29 ^a^	0.23 ± 0.01 ^a^	4.39 ± 0.13 ^a^	617.42 ± 84.40 ^a^
0:100	71.43 ± 28.57 ^a^	0.22 ± 0.01 ^a^	4.56 ± 0.23 ^a^	360.66 ± 245.85 ^a^
***Pseudomonas fluorescens* (PV936737)**				
100:0	95.24 ± 8.25 ^a^	0.23 ± 0.00 ^a^ ^b^	4.39 ± 0.01 ^a^	625.85 ± 48.91 ^a^
80:20	95.24 ± 8.25 ^a^	0.23 ± 0.01 ^a^ ^b^	4.36 ± 0.14 ^a^	535.45 ± 98.88 ^a^
60:40	95.24 ± 8.25 ^a^	0.23 ± 0.00 ^a^ ^b^	4.36 ± 0.04 ^a^	593.97 ± 122.53 ^a^
40:60	85.71 ± 0.00 ^a^	0.23 ± 0.00 ^a^ ^b^	4.37 ± 0.05 ^a^	499.18 ± 49.44 ^a^
20:80	80.95 ± 21.82 ^a^	0.22 ± 0.01 ^a^	4.49 ± 0.14 ^a^	442.79 ± 156.72 ^a^
10:100	85.71 ± 14.29 ^a^	0.23 ± 0.01 ^b^	4.28 ± 0.11 ^a^	513.54 ± 200.22 ^a^
0:100	90.48 ± 8.25 ^a^	0.22 ± 0.01 ^a^	4.52 ± 0.22 ^a^	464.81 ± 88.21 ^a^
***Enterobacter hormaechei* (PV936738)**				
100:0	94.44 ± 9.62 ^a^ ^b^	0.22 ± 0.00 ^a^	4.50 ± 0.04 ^a^	539.44 ± 120.90 ^a^
80:20	100 ± 0.00 ^a^	0.23 ± 0.00 ^a^	4.40 ± 0.06 ^a^	533.81 ± 78.26 ^a^
60:40	95.24 ± 8.25 ^a^ ^b^	0.23 ± 0.00 ^a^	4.37 ± 0.03 ^a^	568.30 ± 53.75 ^a^
40:60	80.95 ± 8.25 ^b^	0.22 ± 0.01 ^a^	4.48 ± 0.16 ^a^	350.00 ± 58.23 ^a^
20:80	90.48 ± 16.50 ^a^ ^b^	0.23 ± 0.01 ^a^	4.43 ± 0.20 ^a^	519.80 ± 264.15 ^a^
10:100	76.19 ± 8.25 ^b^	0.23 ± 0.01 ^a^	4.34 ± 0.15 ^a^	449.73 ± 68.05 ^a^
0:100	80.95 ± 16.50 ^b^	0.22 ± 0.00 ^a^	4.48 ± 0.10 ^a^	479.52 ± 237.38 ^a^
***Pseudomonas aeruginosa* (PV936739)**				
100:0	81.90 ± 3.30 ^a^	0.23 ± 0.01 ^a^	4.27 ± 0.14 ^a^ ^b^	492.29 ± 77.46 ^a^
80:20	83.02 ± 2.87 ^a^	0.24 ± 0.01 ^a^	4.23 ± 0.21 ^a^ ^b^	454.03 ± 156.27 ^a^
60:40	76.19 ± 8.25 ^a^	0.23 ± 0.01 ^a^	4.36 ± 0.11 ^a^ ^b^	409.59 ± 75.79 ^a^
40:60	90.48 ± 8.25 ^a^	0.23 ± 0.01 ^a^	4.35 ± 0.13 ^a^ ^b^	531.84 ± 70.18 ^a^
20:80	87.78 ± 10.72 ^a^	0.23 ± 0.00 ^a^	4.29 ± 0.07 ^a^ ^b^	475.11 ± 170.73 ^a^
10:100	80.95 ± 21.82 ^a^	0.22 ± 0.01 ^b^	4.56 ± 0.12 ^b^	434.63 ± 148.09 ^a^
0:100	90.48 ± 16.50 ^a^	0.23 ± 0.01 ^a^	4.38 ± 0.17 ^a^ ^b^	590.46 ± 245.81 ^a^

Within columns, values with different and same superscript letters represent differences that are significantly and not significantly different, respectively (p < 0.05). Dilution ratios connote inoculum: water ratio.

In presence of *P. aeruginosa* (PV936735) seeds, % germination was highest at 100% inoculum (100%), with decrease at intermediate dilutions (72.22% at 60:40) and fluctuations at other dilutions. Vigor index however showed reduction at mid-level dilutions like 40:60 ( Tables 4 and 16).

In the case of the *P. aeruginosa* (PV936736), % germination was relatively high (66–90%) across dilutions. Vigor index was relatively and showed reduction at 40:60 dilution ( Table 16).

The *P. fluorescens* (PV936737) revealed consistently high % germination (80–95%) across the dilutions. However, germination rate and time remained stable while vigor index varied across dilutions but with no obvious trend ( Table 16).

The *E. hormaechei* (PV936738) treated high % germination (80–100%) across the respective dilutions. However, significant reduction in vigor index was observed at 40:60 dilution. For treatment with *P. aeruginosa* (PV936739), consistent % germination was observed while decreases in vigor index were observed at mid dilutions like 20:80 ( Table 16).

***Cucumber***

Generally, the cucumber seeds showed high germination across the respective inoculum concentrations. However, vigor index showed sharp decline at specific dilutions (especially 20:80) in presence of some of the strains.

In the presence of *P. aeruginosa* (PV936735), high % germination was recorded (75–100%) across the dilutions with no observed significant differences while vigor index was relatively moderate, showing slight decreases at 20:80.

For the *P. aeruginosa* (PV936736) treated seeds, 100% germination was recorded consistently at all the dilutions. Vigor index was generally high across all dilutions with highest value recorded at 100% inoculum (1338) with minute decrease at 60:40.

When treated with *P. fluorescens* (PV936737), % germination stayed nearly at 100% at the different dilutions with the exception at 20:80 where 62.5% was recorded. Vigor index however showed remarkable reduction at 20:80 dilution (204).

For seeds treated with *E. hormaechei* (PV936738), consistently 100% germination was recorded across the respective dilutions while vigor index values were remarkably high and consistent across the respective dilutions, except at 20:80.

In the case of *P. aeruginosa* (PV936739), germination was also observed to be consistently high (87–100%) at the different dilutions while vigor index showed sharp decrease at 20:80 (377). No significant decrease in vigor was observed at other dilutions ( Table 17).

**Table 17.** Effect of inoculum concentration on phytotoxicity of the cucumber seeds in presence of the test bacterial strains.

Inoculum dilution	% germination	Germination rate	Germination time	Vigor index
***Pseudomonas aeruginosa* (PV936735)**				
100:0	83.33 ± 23.57 ^a^	0.23 ± 0.00 ^a^	4.26 ± 0.04 ^a^	866.11 ± 429.76 ^a^
80:20	100 ± 0.00 ^a^	0.22 ± 0.02 ^a^	4.65 ± 0.41 ^a^	940.00 ± 325.27 ^a^
60:40	83.33 ± 23.57 ^a^	0.24 ± 0.01 ^a^	4.16 ± 0.22 ^a^	899.17 ± 142.60 ^a^
40:60	75.00 ± 35.36 ^a^	0.22 ± 0.01 ^a^	4.65 ± 0.11 ^a^	760.00 ± 519.72 ^a^
20:80	75.00 ± 35.36 ^a^	0.22 ± 0.00 ^a^	4.50 ± 0.06 ^a^	523.75 ± 100.76 ^a^
10:100	100 ± 0.00 ^a^	0.23 ± 0.01 ^a^	4.42 ± 0.27 ^a^	1135.00 ± 31.82 ^a^
0:100	100 ± 0.00 ^a^	0.22 ± 0.01 ^a^	4.65 ± 0.29 ^a^	826.25 ± 104.30 ^a^
***Pseudomonas aeruginosa* (PV936736)**				
100:0	100 ± 0.00 ^a^	0.24 ± 0.01 ^a^	4.09 ± 0.22 ^a^	1338.33 ± 30.64 ^a^ ^b^
80:20	100 ± 0.00 ^a^	0.23 ± 0.01 ^a^	4.39 ± 0.23 ^a^	1170.83 ± 189.74 ^a^ ^b^
60:40	100 ± 0.00 ^a^	0.23 ± 0.00 ^a^	4.44 ± 0.09 ^a^	765.00 ± 473.76 ^b^
40:60	100 ± 0.00 ^a^	0.23 ± 0.01 ^a^	4.34 ± 0.16 ^a^	1130.00 ± 28.28 ^a^ ^b^
20:80	100 ± 0.00 ^a^	0.23 ± 0.01 ^a^	4.43 ± 0.10 ^a^	1147.50 ± 49.50 ^a^ ^b^
10:100	100 ± 0.00 ^a^	0.21 ± 0.00 ^b^	4.67 ± 0.08 ^b^	1021.25 ± 178.54 ^a^ ^b^
0:100	100 ± 0.00 ^a^	0.21 ± 0.01 ^b^	4.76 ± 0.27 ^b^	858.75 ± 121.98 ^a^ ^b^
***Pseudomonas fluorescens* (PV936737)**				
100:0	100 ± 0.00 ^a^	0.24 ± 0.01 ^a^	4.26 ± 0.15 ^a^	1264.58 ± 17.09 ^a^
80:20	100 ± 0.00 ^a^	0.23 ± 0.01 ^a^	4.38 ± 0.32 ^a^	1128.75 ± 15.91 ^a^
60:40	100 ± 0.00 ^a^	0.22 ± 0.00 ^a^	4.59 ± 0.31 ^a^	956.25 ± 235.11 ^a^ ^b^
40:60	87.50 ± 17.68 ^a^	0.22 ± 0.01 ^a^	4.55 ± 0.13 ^a^	817.81 ± 289.47 ^a^ ^b^
20:80	62.50 ± 17.68 ^a^	0.23 ± 0.01 ^a^	4.41 ± 0.13 ^a^	204.38 ± 289.03 ^c^
10:100	100 ± 0.00 ^a^	0.22 ± 0.01 ^a^	4.47 ± 0.34 ^a^	967.50 ± 318.20 ^a^ ^b^
0:100	75.00 ± 35.36 ^a^	0.22 ± 0.01 ^a^	4.55 ± 0.37 ^a^	412.50 ± 583.36 ^b^ ^c^
***Enterobacter hormaechei* (PV936738)**				
100:0	100 ± 0.00 ^a^	0.23 ± 0.01 ^a^	4.30 ± 0.10 ^a^	1193.33 ± 80.14 ^a^
80:20	100 ± 0.00 ^a^	0.23 ± 0.02 ^a^	4.32 ± 0.30 ^a^	1038.75 ± 139.65 ^a^
60:40	100 ± 0.00 ^a^	0.21 ± 0.03 ^a^	4.74 ± 0.60 ^a^	1113.75 ± 79.55 ^a^
40:60	100 ± 0.00 ^a^	0.23 ± 0.01 ^a^	4.28 ± 0.25 ^a^	1175.00 ± 77.78 ^a^
20:80	100 ± 0.00 ^a^	0.23 ± 0.01 ^a^	4.43 ± 0.10 ^a^	763.75 ± 606.34 ^a^
10:100	100 ± 0.00 ^a^	0.23 ± 0.01 ^a^	4.42 ± 0.27 ^a^	940.00 ± 205.06 ^a^
0:100	87.50 ± 17.68 ^a^	0.22 ± 0.01 ^a^	4.65 ± 0.29 ^a^	812.92 ± 57.16 ^a^
***Pseudomonas aeruginosa* (PV936739)**				
100:0	100 ± 0.00 ^a^	0.24 ± 0.03 ^a^	4.16 ± 0.49 ^a^	1261.25 ± 146.72 ^a^
80:20	87.50 ± 17.68 ^a^	0.24 ± 0.00 ^a^	4.25 ± 0.02 ^a^	888.44 ± 384.05 ^a^
60:40	100 ± 0.00 ^a^	0.23 ± 0.00 ^a^	4.28 ± 0.06 ^a^	981.67 ± 388.91 ^a^
40:60	87.50 ± 17.68 ^a^	0.23 ± 0.01 ^a^	4.31 ± 0.27 ^a^	660.00 ± 265.17 ^a^
20:80	87.50 ± 17.68 ^a^	0.22 ± 0.00 ^a^	4.48 ± 0.05 ^a^	377.50 ± 176.78 ^b^
10:100	100 ± 0.00 ^a^	0.23 ± 0.00 ^a^	4.41 ± 0.07 ^a^	1032.50 ± 10.61 ^a^
0:100	83.33 ± 23.57 ^a^	0.22 ± 0.00 ^a^	4.57 ± 0.10 ^a^	685.28 ± 324.88 ^a^ ^b^

Within columns, values with different and same superscript letters represent differences that are significantly and not significantly different, respectively (p < 0.05). Dilution ratios connote inoculum: water ratio.

### 3.4 Indole acetic acid production

In the presence of all the bacterial strains, significantly higher IAA production was observed in the presence of tryptophan. In the absence of tryptophan, only minute production of IAA was observed in presence of the strains. This observation was consistent for all the strains and incubation period. In addition, the temporal pattern revealed IAA accumulation during the lag period with consistent increase in mid-log and maximum production in late-log to stationary phase of growth. A comparison among the strains revealed production in presence of tryptophan to be *P. aeruginosa* (PV936735) > *P. aeruginosa* (PV936736) > *P. aeruginosa* (PV936739) > *P.* *fluorescens* (PV936737) > *Enterobacter hormaechei* (PV936738) ( Figures 1- 5).

**Figure 1.**
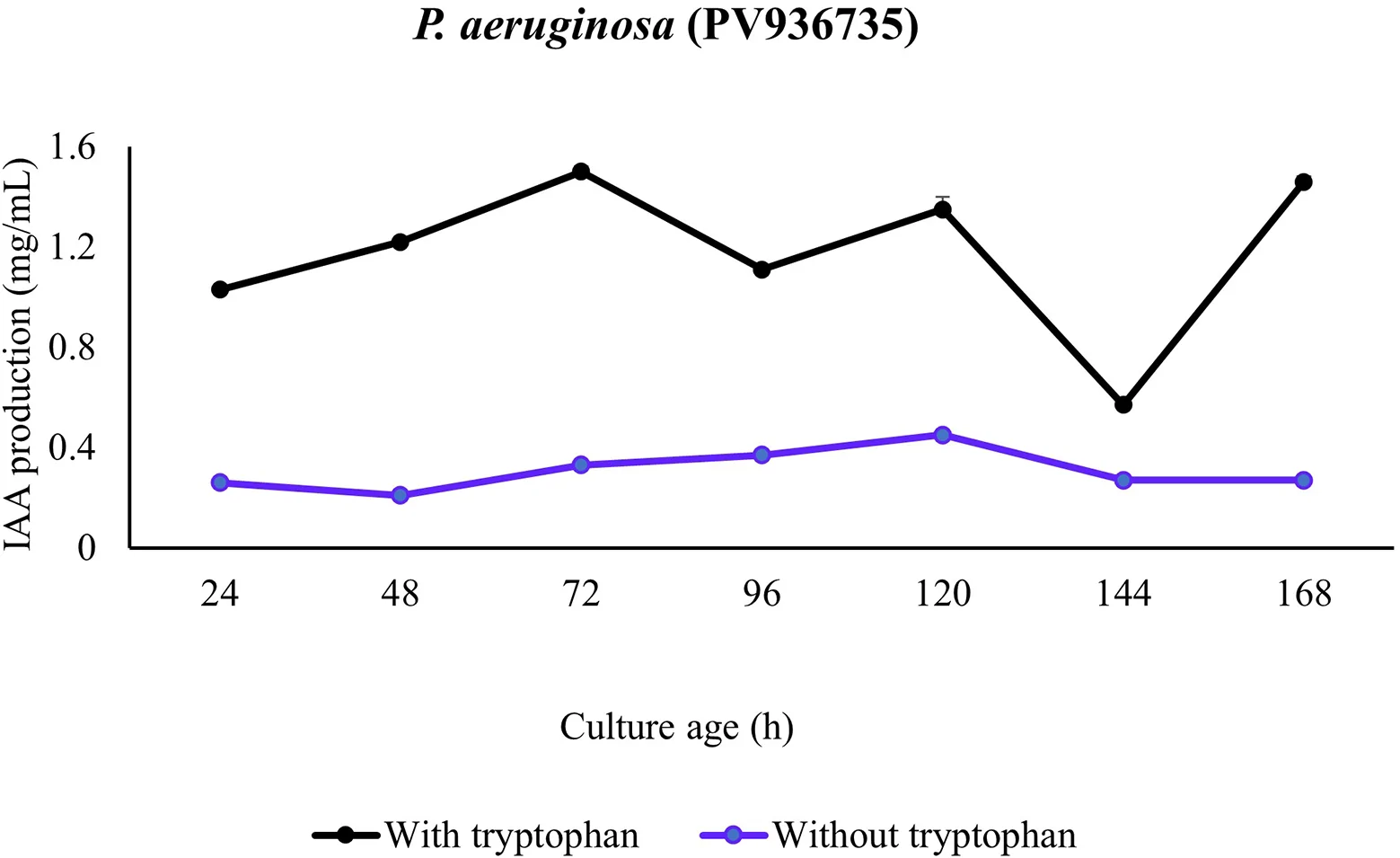
IAA production in presence and absence of tryptophan by *P. aeruginosa* (PV936735) during the growth period.

**Figure 2.**
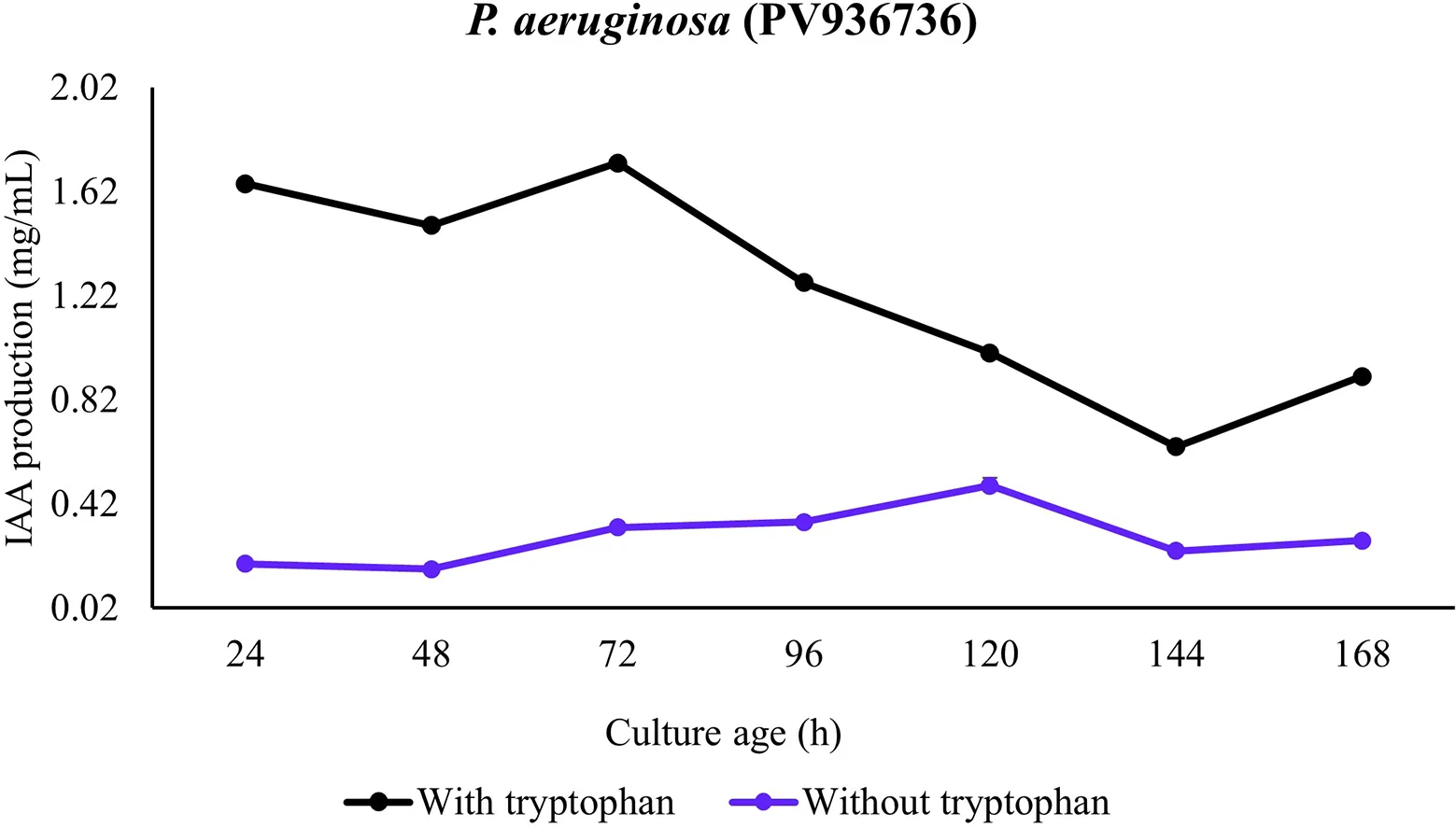
IAA production in presence and absence of tryptophan by *P. aeruginosa* (PV936736) during the growth period.

**Figure 3.**
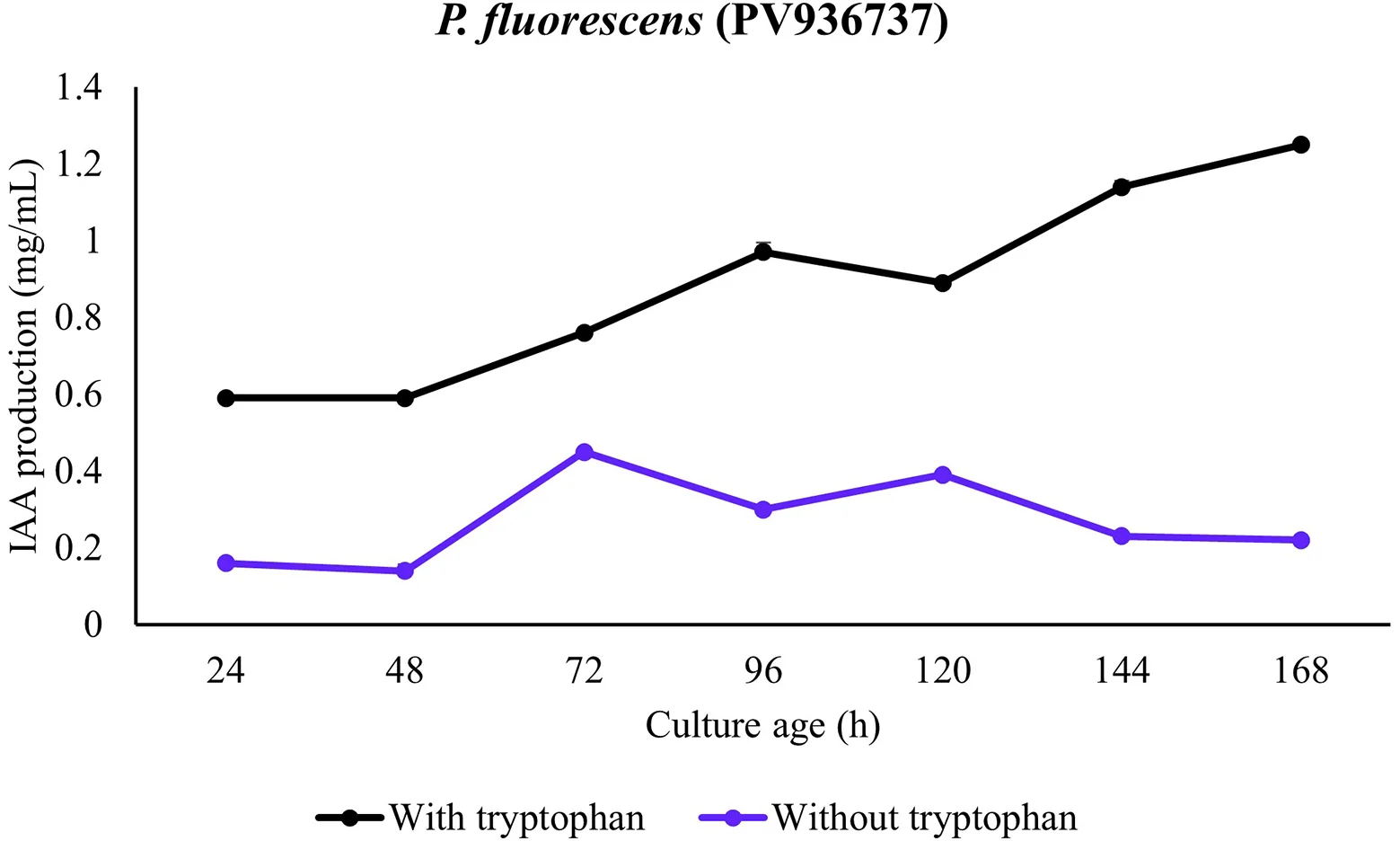
IAA production in presence and absence of tryptophan *P. fluorescens* (PV936737) during the growth period.

**Figure 4.**
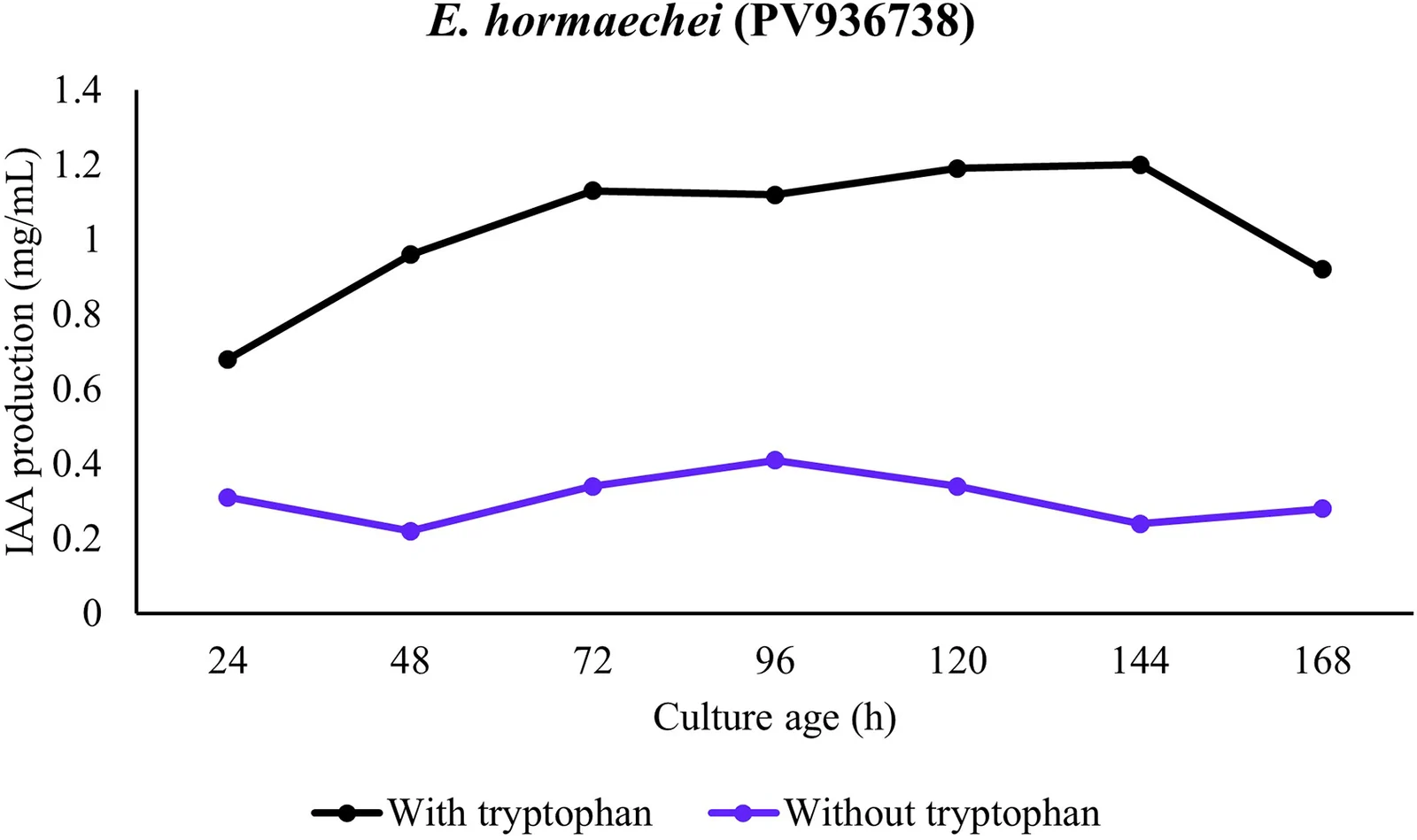
IAA production in presence and absence of tryptophan *E. hormaechei* (PV936738) during the growth period.

**Figure 5.**
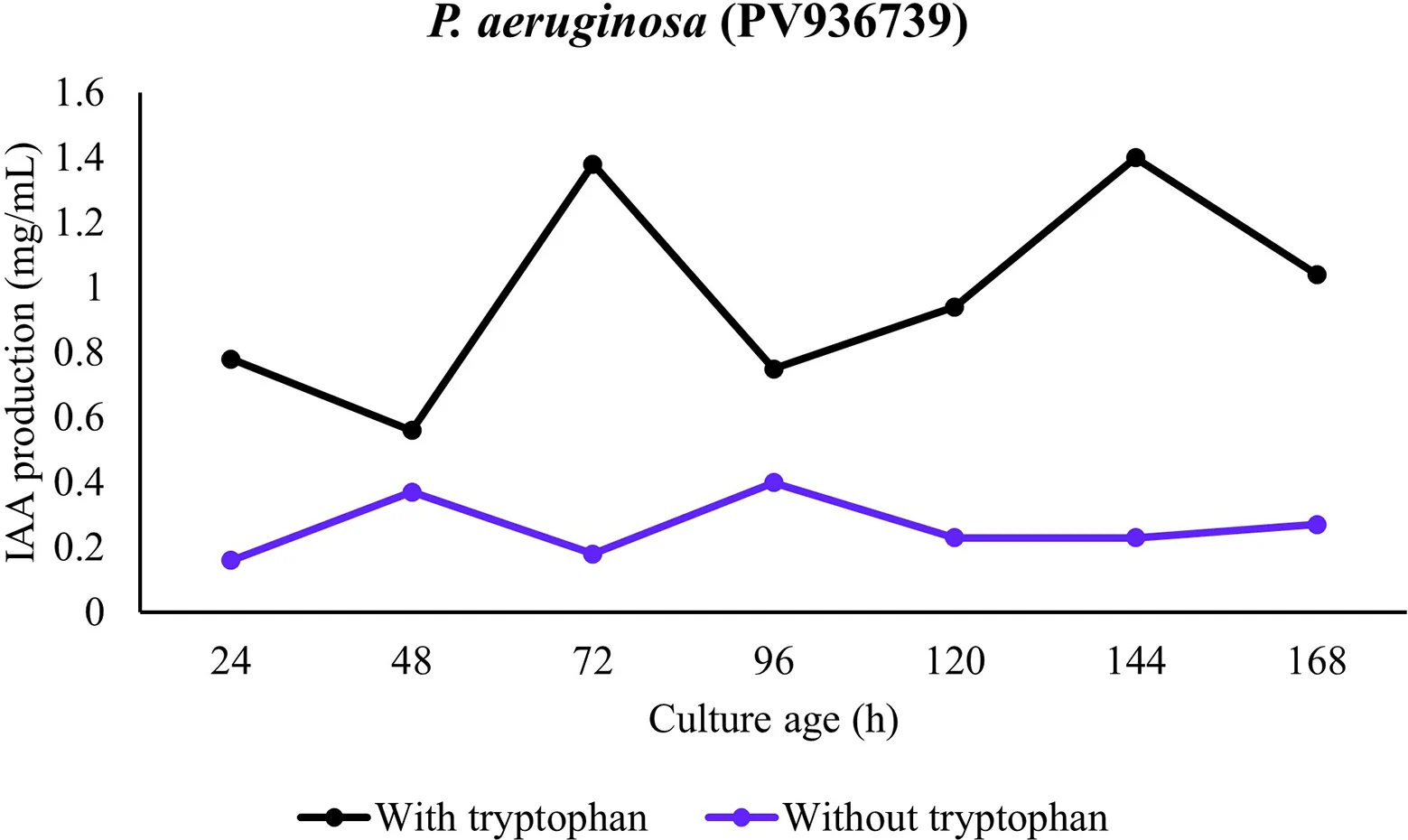
IAA production in presence and absence of tryptophan *P. aeruginosa* (PV936739) during the growth period.

For *P. aeruginosa* (PV936735), IAA production in the presence of tryptophan showed consistent increase from low level during initial growth to peak level at late log/early stationary phase. Indole acetic acid concentration during initial growth phase. A similar pattern was followed in the absence of tryptophan, although values were significantly lower than for tryptophan-supplemented culture ( Figure 1).

In the case of *P. aeruginosa* (PV936736), a similar pattern as observed for *P. aeruginosa* (PV936735) was recorded rising from low (lag phase) to rapid rise (mid log phase) to peak level (stationary phase). However, in culture without supplemented tryptophan, IAA production was only at baseline level with no remarkable rise with time of incubation ( Figure 2).

In medium supplemented with tryptophan, IAA production by *P. fluorescens* (PV936737) increased from mid-log and to highest levels in early to mid-stationary phase. The increase in IAA concentration was however lower observed to be relatively moderate, when compared with *P. aeruginosa.* When tryptophan was absent, only minimal IAA was produced showing small increase in stationary phase only ( Figure 3).

The observed trend in IAA production in the presence of *E. hormaechei* (PV936738) revealed remarkable increase from late-log and to highest concentration in stationary phase. When tryptophan was absent in culture, only baseline IAA was produced. However, a modest late rise in stationary phase was observed, which was evidently higher than production in presence of the other strains ( Figure 4).

Also, in presence of *P. aeruginosa* (PV936739), although a start to IAA production was observed in stationary phase in tryptophan-supplemented culture, a sharp increase at late-log/early-stationary was recorded. When tryptophan was absent, IAA production was however observed to be minute throughout the period of incubation ( Figure 5).

## 4. Discussion

In this study, three rhizobacterial species ( *Pseudomonas aeruginosa*, *Pseudomonas fluorescens*, and *Enterobacter hormaechei*) were used. These bacterial species have been implicated in the production of phytohormones, hydrogen cyanide and ammonia, which are major secondary metabolites produced by deleterious rhizobacteria ( Lakshmi *et al., *2015; Khianngam *et al., *2023). Deleterious rhizobacteria are predominantly saprophytic bacteria that aggressively colonise plant seeds, roots and rhizosphere. They feed on organic compounds secreted by plant root cells known as exudates ( Fang *et al., *2022). The soil environment is made up of high population of both beneficial and deleterious microorganisms known as rhizobacteria ( Kremer, 2006).

Antibacterial potentials of the test rhizobacterial strains showed varying inhibitory activity against several pathogens. *Pseudomonas aeruginosa* (PV936735) showed broad spectrum inhibition, producing clear zones of inhibition against *Bacillus proteolyticus*, *Xanthomonas campestris*, *Klebsiella pneumoniae*, *Pseudomonas aeruginosa* and *Pseudomonas fuscoginae.* This observation is in line with the identified characteristics of *Pseudomonas aeruginosa* as a strong producer of diffusible volatile organic, compounds such as rhamnolipids, pyocyanin and phenazines that suppress bacteria growth ( Si *et al., *2024). This corroborates with existing findings of antibiotic effect exhibited by rhamnolipid metabolite ( Vatsa *et al., *2010). Another study by Kanthaiah & Velu, (2022) characterized the bioactive metabolite from *Pseudomonas aeruginosa* VRKK1 and exploited its antibacterial behavior against *Xanthomonas campestris* a causative agent of bacterial blight disease in cowpea. Akpor *et al., *(2023) reported in a study on the growth inhibitory potential of metabolites produced by *Pseudomonas* and *Bacillus* species that growth of *Salmonella typhi* and *Klebsiella pneumoniae* were inhibited during the period of incubation. In another study conducted by Bharali *et al., *(2014), the antimicrobial activity of *Pseudomonas aeruginosa* was tested against *Klebsiella pneumoniae, Bacillus subtilis*, *Escherichia coli* and *Staphylococcus aureus*, and the report showed antibacterial activity against all tested pathogenic strains except *Staphylococcus aureus.*

The *Pseudomonas fluorescens* (PV936737) used in this study showed inhibition against *Bacillus subtilis* (25 mm) and *Klebsiella pneumonia* (20 mm). This result corresponds with the study conducted by Sharma *et al., *(2019) that crude metabolite extracts of *P. fluorescens* showed inhibition against *Klebsiella pneumonia* (18.67) and Bacillus subtilis (25.67 mm).

A wide zone of inhibition against *Bacillus subtilis* by *Enterobacter hormaechei* (PV936738) was observed to be the most remarkable across all test bacterial isolates. Zhang *et al., *(2024) have reported that *Enterobacter* species isolated from the *Zea mays* rhizosphere have broad spectrum antibacterial activity due to the ability to secrete lipopeptides. It is reported that *Enterobacter hormaechei* is an opportunistic pathogen possessing deleterious potential towards both plants and human ( Raza *et al., *2022).

The test seeds for the study were primed in the respective rhizobacterial inoculums before planting. Priming of seeds before planting is indicated to help in improving germination. During priming, seeds are indicated to have access to sufficient moisture, which is essential for rapid germination ( Patanè *et al., *2016). In addition, priming hydrates seeds, thus leading to enhancement of vigor and germination rate ( Rhaman *et al., *2020).

The present study revealed enhanced seed germinability when primed with young cultures (24-48 h) of the test bacterial isolates. However, seeds primed with older culture (72 h -168 h) were shown to have deleterious effect on germinability parameters. It has been reported that culture age influences the vigor and germination of seeds and as the bacteria culture ages, their physiology, ability to compete for nutrients and capacity to produce metabolites are altered which interferes with their interaction with seeds during bio-priming ( Srivastava *et al., *2024). In a study by Miteva-Staleleva *et al., *(2025), freshly grown bacterial cultures were reported to generate optimal quantities of phytohormones, hydrogen cyanide, indole-3-acetic acid, siderophores, and enzymes, such as ACC deaminase, as well as other metabolites that stimulate seed germination A similar observation has been reported by Ludueña *et al., *(2018) in a related study. In their report, they indicated that bacterial cultures in their exponential phase secrete phytohormones, such as auxins, gibberellins and cytokinins which stimulate seed germination and development.

In the present study, seeds treated with *P. fluorescens* (PV936737) showed the highest% germination followed closely by *E. hormaechei* (PV936738) and *P. aeruginosa* (PV936739). However, cowpea seeds treated with *P. aeruginosa* (PV936735) were observed to have the lowest% germination. Isolates incubated beyond 120 h negatively affected seed germination, suppressed seedling vigor and decreased germination, whereas cultures with shorter incubation periods enhanced germination percentage, vigor, and germination rate in *Pseudomonas aeruginosa* (PV936735), *Pseudomonas aeruginosa* (PV936736), *Pseudomonas fluorescens* (PV936737), and *Pseudomonas aeruginosa* (PV936739). It is opined that older bacterial cultures accumulate high concentrations of secondary metabolites, which inhibits seedling germination and plant vigor ( Hasan *et al., *2024). Although the sorghum seeds showed high% germination when primed in the respective culture ages, significantly lower reduced vigor were recorded in seeds primed in older cultures. In a related investigation, *Pseudomonas* species were reported to enhance plant growth when in their active metabolic phase, however, under unfavorable or nutrient-limiting conditions, they may secrete toxic secondary metabolites that inhibit growth ( Mehmood *et al., *2023). In addition, Katiyar *et al., *(2025) observed that extended incubation of *Pseudomonas aeruginosa* cells led to hydrogen cyanide buildup, which caused reduced vigor and limited root elongation in legumes.

In this study, *Enterobacter hormaechei* (PV936738) maintained high germination percentage and vigor index values across all culture ages, suggesting its reduced phytotoxic effects on the test seeds. This report aligns with the study done by Agboola *et al., *(2023), which identified that *Enterobacter hormaechei* produces moderate quantities of indole-3-acetic acid and phosphate-solubilizing enzymes that improved the germination and early growth of maize and cowpea seedlings.

At the respective steeping periods of the test seeds in the inoculums, although no consistent pattern of increase or decrease were noticed for the germinability parameters assessed, generally longer steeping periods reduced germinability of the cowpea, maize and soybean seeds. A similar observation has been reported by earlier investigator ( Ajinde *et al., *2023). According to Ajinde *et al., *(2023), prolonged steeping reduced the final germination percentage of cowpea and soybean seeds due to over-imbibition, which made the seeds soggy and slowed germination. Similarly, Basavaraj *et al., *(2019) found in a study on pearl millet that longer steeping duration encouraged bacterial buildup on the seed surface, which resulted in reduced seed energy efficiency since more energy was used in sustaining bacterial growth. In another study, Teixeira *et al., *(2021) reported that treating soybean seeds with *Bacillus valezensis* (CMRP 4490) improved germination performance, while Pooja *et al., *(2021) discovered that treating chickpea seeds with *Bacillus valezensis* (MNB08) improved the final germination percentage.

In this study, remarkably reduced percent germination and vigor were recorded for sorghum seeds at all treatments at 1 h steeping duration. This however contradicts the theory that over-imbibition reduces final germination. A study by Ituen *et al., *(1986) reported that sorghum seed took 48 h to reach its full water absorption potential, showing the impermeability of the seed. Another study by Teshome *et al., *(2018) reported increase in final percent germination as the steeping duration increased for almost all varieties of sorghum seeds tested against all priming treatments. The reduced vigor and germination observed at short steeping duration may be due to the inability of the inoculums to penetrate the sorghum. For the cucumber seeds, priming in *Pseudomonas aeruginosa* (PV936735), *Pseudomonas fluorescens* (PV936737) and *Pseudomonas aeruginosa* (PV936739) at longer steeping time significantly reduced germination percentage and vigor index.

The present study revealed that higher inoculum concentration exhibited phytotoxic effects on germinability of some of the seeds. Ajinde *et al., *(2023) have reported that concentration of the bacterial treatment used in bio-priming affects the seed germinability parameters ( Ajinde *et al., *2023). For the cowpea seeds treated with high concentrations of *Pseudomonas aeruginosa* (PV936735), *Pseudomonas aeruginosa* (PV936736), *Pseudomonas fluorescens* (PV936737) and *Pseudomonas aeruginosa* (PV936739), reduced germination and vigor index were observed. This might be as a result of accumulation of toxic metabolites due to the elevated bacterial loads indicating deleterious effect of high concentration of inoculum. Maize seeds showed slight sensitivity to inoculum concentration, with high inoculum occasionally reducing seed performance. Only slight sensitivity to inoculum concentration was observed for percentage germination while vigor index was negatively affected at certain dilutions. According to a study by Chahtane *et al., *(2018), the presence of *Pseudomonas aeruginosa* inhibited *Arabidopsis* seeds germination.

Overall, it was observed that the soybean seeds in this study were tolerant to varying inoculum concentrations, with no obvious negative effects on the parameters of germinability while sorghum seeds were relatively sensitive to the concentration of inoculum applied in the treatment of the seed, particularly in the observed changes in the vigor index where the values decreased with intermediate dilution with high percentage germination of the seeds. For the cucumber seeds, high percent germination was observed at the different inoculum concentration-treatments, however, vigor index showed sharp decline at specific dilutions in the presence of some test bacterial treatments. In a related study by Galelli *et al., *(2024), tomato seeds treated with a known concentrations of *Bacillus subtilis* biofilm showed improved root elongation and germination rates. Existing research on seed biopriming and inoculation showed that although the percentage of germination was often relatively high, the vigor index, seedling development, and the overall seed quality can be significantly different based on the inoculum concentration and the duration of the treatment ( Chen *et al., *2021).

In this study, significantly higher IAA production was reported in presence tryptophan by all the test rhizobacterial strains while only minute production was observed in the absence of tryptophan. This observation was consistent for all the strains and incubation period. Indole-3-acetic acid is a member of the group of phytohormones and is a product of L-tryptophan metabolism in microorganisms. It is generally considered as the most import native auxin in plant growth and functions as an important signaling molecule in the regulation of plant development. The significantly reduced IAA produced in medium without supplemented tryptophan as observed in presence of the test isolates used in this study has been reported by earlier investigators ( Lata *et al., *2024).

In a previous study carried out by Khare and Arora (2010), *Pseudomonas aeruginosa* was observed to produce indole acetic acid, corroborating the report of this study. In this study, *Pseudomonas aeruginosa* (PV936735) and *Pseudomonas aeruginosa* (PV936736), indole acetic acid production in the presence of tryptophan showed consistent increase from low level during initial growth to peak level at stationary phase. However, *Pseudomonas aeruginosa* (PV936736) showed no remarkable rise with incubation time in cultures without supplemented tryptophan. In a related study, *Pseudomonas* isolates obtained from the rhizosphere of chickpea ( *Cicer arietinum L.)* and green gram ( *Vigna radiata*) were found to produce significant amount of IAA when grown in medium broth supplemented with L-tryptophan ( Malik and Sindhu, 2011). In this study, indole acetic acid production by *P. fluorescens* (PV936737) in medium supplemented with tryptophan increased from mid-log to highest levels in early to mid-stationary phase but when tryptophan was absent, only minimal IAA was produced showing small increase in stationary phase. Similar results were obtained when cuttings of sour cherry ( *Prunus cerasus*) and black-currant ( *Ribes nigrum*) were inoculated with a recombinant strain of *Pseudomonas fluorescens* that produced increased amount of IAA ( Dubeikovsky *et al., *1993; Malik and Sindhu, 2011).

*Enterobacter hormaechei* was examined as plant growth-promoting bacteria with IAA-producing potential for improvement in the growth of *Lycopersicum esculentum* in a study by Ranawat *et al., *(2021). Indole acetic acid production in presence of *Enterobacter hormaechei* (PV936738) revealed remarkable increase from late-log to highest concentration in stationary phase.

In this present study, all bacterial strains produced peaked levels of indole-3-acetic acid production between the late-log to stationary phases of growth, corresponding to 72–168 h of incubation. This is consistent with the study of Baggam *et al., *(2017). The effects of incubation duration on IAA production had varying reports. Kumar *et al., *(2021) reported that the highest level of indole-3-acetic acid was produced after 72 h of incubation. This observation is however contrary to the results by Datta and Basu (2000) and Kumari *et al., *(2018), who reported that the duration of incubation beyond 24 hours did not show any significant improvement in the production of IAA, which implies that the synthesis was at its peak in the initial growth phase of the culture. The variations in incubation time in studies could be explained by the differences in the availability of nutrients and oxygen. A previous study conducted by Hussain *et al., *(2015) discovered that keeping inoculums for longer incubation durations may enhance accumulation of IAA due to higher metabolic activity and biomass formation.

In a related study, Lata *et al., *(2024) recorded that IAA-producing *Pseudomonas* isolates had an initial adverse effect by causing stunted root and shoot growth in chickpea seeds. A similar observation was recorded when sour cherry ( *Prunus cerasus*) and black currant ( *Ribes nigrum*) cuttings were inoculated with a recombinant strain of *Pseudomonas fluorescens* that produced higher concentration of indole-3-acetic acid. Inoculation of cherry cuttings with high bacterium density on the roots inhibited the growth of roots, whereas low density on black-currant stimulated growth ( Barazani and Friedman, 2023). In a different study, Syed *et al., *(2023) indicated that *Pseudomonas fluorescens* that produces siderophore promoted the growth of chickpea seeds.

## 5. Conclusion

In this study, rhizobacteria with deleterious potentials were successfully isolated, identified and functionally characterized. Their inhibitory effects were validated across multiple test crops using germination and vigor metrics. The phytotoxicity of isolates was dependent on culture age, steeping time and inoculum concentration.

In this study, older inoculum ages of respective rhizobacterial strains showed phytotoxic effect on seedling vigor and percentage germination whereas younger cultures with shorter incubation periods improved seed germinability. *Pseudomonas aeruginosa* (PV936735) and *Pseudomonas aeruginosa* (PV936736) consistently showed culture age-dependent phytotoxicity across the seeds revealing accumulation of inhibitory metabolites as the cultures aged.

With regards to the respective steeping period adopted in this study, no consistent pattern of increase or decrease was observed for the germinability parameters assessed. For cowpea, maize and soybean seeds, generally longer steeping period was observed to reduce the seedling germinability which suggested that prolonged steeping of seeds in inoculum enhanced the penetration of inhibitory compounds. High concentrations of the inoculums used for steeping the seeds in this study were observed to have phytotoxic impacts on seed germinability. This however is likely to be as a result of accumulation of toxic metabolites due to increased bacterial loads in inoculums with high bacteria concentration.

This study also showed that the production of indole acetic acid (IAA) by the rhizobacterial strains was significantly influenced by the presence or absence of tryptophan and incubation period. High IAA production was observed for all the rhizobacterial strains when supplemented with tryptophan while significantly reduced IAA production was observed in the absence of tryptophan. With regards to incubation period, increased level of IAA production was observed with longer incubation period which might suggest increased accumulation due to high metabolic activity.

## Data Availability

All dataset used for this manuscript are available at: Figshare: **Phytotoxicity and indole acetic production by rhizobacterial strains**. https://doi.org/10.6084/m9.figshare.30847679.v2 ( Akpor and Abiodun, 2025). This project contains the following data:
• Indole acetic acid production • Phytotoxicity_incubation duration effect • Phytotoxicity_inoculum concentration effect • Phytotoxicity_steeping duration effect • Agarose gel elctrophersis of the amplified fragments of the isolates Data are available under the terms of the Creative Commons Zero "No rights reserved" data waiver (CC0 1.0 Public domain dedication).
