# Supplementary material for: Phytotoxicity and Indole Acetic Production by Rhizobacterial Strains
Source: F1000Res. 2026 Jul 15;15:45. Originally published 2026 Jan 10. [Version 2] doi: 10.12688/f1000research.174474.2 (PMC13760145; doi:10.12688/f1000research.174474.2)
Supplement: Peer review history

# Reviewer report: Reviewer response for version 2

**DOI:** 10.5256/f1000research.204673.r532180

**Published:** 2026-11-12

**Authors:** Muhammad Abrar

**Affiliations:** 1 Lanzhou University, Lanzhou, China

The manuscript titled “Phytotoxicity and Indole Acetic Production by Rhizobacterial Strains” reports the isolation and characterization of rhizobacterial strains exhibiting phytotoxic and antimicrobial activities. The findings demonstrate that the inhibitory effects of these isolates on seed germination and seedling vigor vary with culture age, steeping duration, and inoculum concentration. Furthermore, all isolates produced measurable IAA, suggesting a potential association between metabolite production and phytotoxic effects, highlighting their potential for further investigation as bioherbicide candidates. Overall, the study addresses an interesting and potentially valuable research topic. However, the manuscript is poorly written and presented, with substantial improvements needed in its organization, scientific clarity and interpretation of the findings. Additionally, the entire manuscript should undergo thorough English language editing to correct grammatical errors, improve sentence structure and enhance overall readability.

 It is important to include continuous line numbering throughout the manuscript to facilitate the peer-review process. This will help reviewers identify specific sections and provide precise comments, making it easier for the authors to locate the relevant passages and implement the necessary revisions.

 Consider revising the title to make it more engaging, informative and specific. The current title reads more like a general statement than a focused research title. A good manuscript title should be concise yet informative, clearly reflecting the main theme and key focus of the study. It should allow readers to understand the central message and scope of the research at a glance.

 Please format the authors’ affiliations according to the journal’s guidelines. Most journals require numerical or alphabetical superscripts after each author’s name to indicate their respective affiliations. This convention should be followed consistently, even when all authors share the same institutional affiliation.

**Abstract**

 The abstract requires further improvement to establish a clear rationale and better communicate the novelty and significance of the study. Although the problem statement is reasonably well presented, the specific research gap in current knowledge is not clearly identified. You should explicitly highlight what remains unknown or insufficiently investigated in the existing literature to justify the need for this study. The study rationale and objectives should then be strengthened to demonstrate how your work addresses this knowledge gap, emphasizing its novelty and potential contribution to the field. Furthermore, the methodological description is unclear and requires further clarification. For example, it is not evident whether bacteria were initially screened from five different crops and whether subsequent analyses were performed using the selected bacterial isolates. Please clarify the experimental procedures. The methodology should be sufficiently clear and logically presented so that readers can understand the overall experimental approach without ambiguity. In addition, the results should be presented in a systematic and mechanistic manner rather than reporting individual findings randomly. Organize the results around the study objectives and explain the relationships between different parameters to establish a clear understanding of the mechanisms underlying the observed responses. This systematic presentation will also provide a stronger foundation for drawing meaningful conclusions. Finally, the conclusion should directly reflect the major findings, address the research objectives and remain consistent with the central theme and scope of the manuscript title. Avoid broad or generalized statements that are not adequately supported by the presented results. For example, the current statement, "The indigenous origin of the DRB evaluated in this study enhances environmental compatibility and reduces ecological disturbance risks", does not adequately communicate the key findings or directly address the stated objectives. Please replace such general claims with specific, evidence-based concluding statements that highlight the actual contributions and significance of your research.

**Keywords**

 It is recommended that you revise the keywords to better reflect the specific focus and scientific contributions of your study. Avoid using overly general terms or simply repeating words already included in the manuscript title. Instead, select specific, relevant and scientifically appropriate keywords that capture the key concepts and novelty of your research. Well-selected keywords will improve the manuscript's visibility, indexing and global discoverability, making it easier for interested readers to find your work after publication.

**Introduction**

 The introduction lacks a clear and logical progression of ideas, with insufficient transitions between individual statements and paragraphs. The arguments should be better connected to establish a coherent scientific narrative and guide readers naturally toward the rationale and objectives of the study. A well-structured introduction should begin by introducing the research problem, followed by its impacts on crops and soil in the context of the present study. It should then discuss the existing strategies used to address this problem, critically evaluate their effectiveness and limitations, and subsequently identify the specific research gap in current knowledge. This knowledge gap should provide a clear justification for the present study and naturally lead to the development of its novel objectives and research hypothesis. Additionally, please update the references throughout the manuscript by incorporating recent and relevant literature to support the current state of knowledge. Older references should generally be retained only when citing classical methodologies or universally accepted scientific concepts. This will strengthen the scientific background, improve the relevance of the discussion and better establish the novelty and significance of your work.

**Materials and methods**

 Although the methodology is presented reasonably well, further improvement is needed in its organization and structure. It is recommended that you present the experimental procedures in a clear, logical sequence, following the actual workflow of your study. Group related methodological information under appropriate subsection headings to ensure that similar types of content are presented together. A well-organized methodology will improve readability, facilitate understanding of the experimental design and ensure that the study can be easily reproduced by other researchers.

**Results**

 It is recommended that you present your results in a systematic and logically organized manner to help readers understand the underlying mechanisms responsible for the observed responses. The subsection headings should be concise yet sufficiently informative to communicate the main findings and key message of the content discussed under each heading. Avoid using vague headings that do not clearly indicate the focus of the results. For example, the current first subsection heading, "Rhizobacterial strains", does not adequately convey whether the subsection discusses bacterial isolation, screening, selection or characterization. Please revise this heading to more accurately reflect the specific findings presented in that section. Ensure that all subsection headings are informative and results-oriented. Additionally, consider shortening and streamlining the results section, as its current length may affect readability and reduce reader engagement. Focus on presenting the important findings in the main manuscript and consider moving supplementary or less critical results to the supplementary materials. This will help maintain a clear narrative without overwhelming readers with excessive details. Furthermore, consider presenting selected datasets through appropriate graphical visualizations rather than relying predominantly on tables, as well-designed figures can facilitate data interpretation, highlight important patterns and improve the overall presentation and accessibility of your findings.

**Discussion**

 Please organize the discussion using subsection headings that correspond to those in the results section to maintain consistency and a logical flow throughout the manuscript. The subsection headings should be informative and clearly reflect the main findings or topics being discussed under each section. Within each subsection, briefly summarize the relevant findings before discussing the possible physiological, biochemical or molecular mechanisms underlying the observed responses, supported by relevant literature. Avoid simply repeating the results; instead, focus on interpreting the findings and explaining their scientific significance. Additionally, there is no need to include a separate subsection heading for study limitations. You may briefly acknowledge the key limitations and future research directions in the final paragraph of the discussion or incorporate them into the conclusion section, depending on the journal's guidelines.

Is the work clearly and accurately presented and does it cite the current literature?

Partly

If applicable, is the statistical analysis and its interpretation appropriate?

Yes

Are all the source data underlying the results available to ensure full reproducibility?

Yes

Is the study design appropriate and is the work technically sound?

Yes

Are the conclusions drawn adequately supported by the results?

Partly

Are sufficient details of methods and analysis provided to allow replication by others?

Yes

Reviewer Expertise:

Plant-Microbe-Soil Interactions, Agronomy, Agricultural Microbiology

I confirm that I have read this submission and believe that I have an appropriate level of expertise to confirm that it is of an acceptable scientific standard, however I have significant reservations, as outlined above.

# Reviewer report: Reviewer response for version 2

**DOI:** 10.5256/f1000research.204673.r520892

**Published:** 2026-10-05

**Authors:** Rene Diaz-Herrera

**Affiliations:** 1 Universidad Autónoma de Coahuila, Unidad Saltillo, Mexico

-The title is appropriate; however, it may not fully reflect the scope and content of the manuscript. I recommend considering a slight modification to the title to better encompass the main aspects addressed in the study.

 Introduction:

 1- The punctuation should be carefully reviewed, as several sentences are missing appropriate periods and commas.

 2- Please review the recency of the references cited (Hussain et al., 2015; Ramesh et al., 2017; Heap et al., 2014).

 3- The manuscript currently only mentions the phytotoxic effects of the phytohormones produced by bacteria. It is also important to mention their beneficial effects, as they are relevant to the results presented in this study.

 4- Why did you select those seeds?

 Methodology:

 1- In the equations used to calculate the germination parameters, please specify the units in which the results are expressed.

 Results:

 1- Specifying the units in which the germination parameters are expressed in the tables in the Results section would improve clarity and facilitate the interpretation of the data.

 2- In the indole acetic acid production section, it says "only minute production"

Is the work clearly and accurately presented and does it cite the current literature?

Partly

If applicable, is the statistical analysis and its interpretation appropriate?

Yes

Are all the source data underlying the results available to ensure full reproducibility?

Yes

Is the study design appropriate and is the work technically sound?

Yes

Are the conclusions drawn adequately supported by the results?

Yes

Are sufficient details of methods and analysis provided to allow replication by others?

Yes

Reviewer Expertise:

Agricultural Sciences, Microbiology, and Biotechnology

I confirm that I have read this submission and believe that I have an appropriate level of expertise to confirm that it is of an acceptable scientific standard.

# Reviewer report: Reviewer response for version 2

**DOI:** 10.5256/f1000research.204673.r506499

**Published:** 2026-08-14

**Authors:** Jiban Shrestha

**Affiliations:** 1 National Plant Breeding and Genetics Research Centre, Khumaltar, Nepal

Abstract:

 Authors need to include the location and year of experiment. Provide treatment levels in methods

 Introduction:

 Provide the new insights, specific research gap addressed by this study. Try to include recent more text references. Provide limitation and specific objectives of this study.

 Materials and Methods:

 When and where did you carry out this study?

 Provide equation number for formula used.

 Provide clear information on experimental design and replication. The seed viability test based on floating/sinking seeds is insufficiently reliable; use a standard germination or tetrazolium test.

 Provide actual inoculum concentrations/CFU mL⁻¹

 Results:

 In Table 1, provide unit of measurement (%) in column headings only. Write on footnote what does the 'E value' indicates?

 In Table 2-17 what does the sign (plus minus) indicates for what information, write in footnote of table 2-17. The number of tables used in the manuscript is very large, keep only important 4-5 tables (remove or combine).

 Discussion:

 Do not keep any figures in Discussion section. Keep respective tables or figures number while linking results with discussion information. Provide latest references.

 References:

 All references should follow the journal rules. Provide DOI for journal references.

Is the work clearly and accurately presented and does it cite the current literature?

No

If applicable, is the statistical analysis and its interpretation appropriate?

Partly

Are all the source data underlying the results available to ensure full reproducibility?

No

Is the study design appropriate and is the work technically sound?

No

Are the conclusions drawn adequately supported by the results?

No

Are sufficient details of methods and analysis provided to allow replication by others?

No

Reviewer Expertise:

Plant Breeding, Plant Genetics, Plant Production, Plant Science

I confirm that I have read this submission and believe that I have an appropriate level of expertise to confirm that it is of an acceptable scientific standard, however I have significant reservations, as outlined above.

# Reviewer report: Reviewer response for version 1

**DOI:** 10.5256/f1000research.192378.r463345

**Published:** 2026-04-11

**Authors:** Sowmyalakshmi Subramanian

**Affiliations:** 1 Department of Plant Sciences, MacDonald Campus, McGill University, Montreal, Québec, Canada

The study focuses on the use of phytotoxic rhizobacteria to control weeds. This is a sustainable way of keeping the weeds under check. This study however has flaws in experimental design and the manuscript is not complete.

 Also, some of the bacteria mentioned in the introduction especially *Pseudomonas aeruginosa*, *Pseudomonas syringae* and *Enterobacter hormaechei* are also cross kingdom infectors. Without proper validation, these should not be even tested for a study such as this.

 Please load a manuscript with lines numbered so that it is easier to review.

 Check the SI units for correct representation. Eg., ml must be mL, etc.

**In the manuscript:** 2. Materials and methods

 2.3 Preliminary phytotoxicity and antimicrobial screening

 Prior to use, test seeds used were surface-sterilized with sodium hypochlorite (5% v/v) solution for 5 min and rinsed three times in distilled water.

**Reviewer comments:** The seeds used in this study were cucumber ( *Cucumis sativus*), soybean ( *Glycine max*), sorghum ( *Sorghum bicolor*), cowpea ( *Vigna unguiculata*) and maize ( *Zea mays*). While sorghum and maize are hardy seeds, and cowpea to an extent, 5 mins of seed sterilization affects the embryo of cucumber and especially soybean profoundly. Why was an universal approach taken to surface sterilize seeds?

**In the manuscript:** For viability testing, approximately 200 surface-sterilized seeds from a lot were placed in a 500 mL-distilled water beaker and observed for floated seeds. The seeds that floated were regarded as non-viable and discarded. Preliminary screening for phytotoxic effect for the bacteria isolates was carried out following the method described by Akpor et al., (2022). The respective seeds were soaked in 24-h-old nutrient broth cultures of the respective test isolates and allowed to stand for 2 h. Seven (7) seeds were withdrawn and planted in Petri dishes containing 3.48 g of cotton wool.

**Reviewer comment: **Were the germination experiments done only with 7 seeds per treatment? If yes, this is not a correct representation for a germination test. The data is highly extrapolated for the results to be taken into consideration. In order to get a germination percentage, at least 100 seeds/treatment have to be considered.

 What was the logic behind soaking 200 seeds but only 7 seeds taken for the germination experiment?

**In the manuscript: **With regard to the effect of inoculum concentration, test seeds were soaked in varying concentrations of a respective inoculums of known culture age and steeped for 2 h before planting and incubation for a 7-d duration. The inoculum dilutions used for steeping were 100:0, 80:20, 60:40, 40:60, 20:80 and 100:0, inoculum to water ratios, respectively.

**Reviewer comment:** There are two 100:0 dilutions. The last one should be 0:100.

**In the manuscript: **3. Results

 3.1 Rhizobacterial strains

 A total of five rhizobacterial strains, which consists of three strains of *Pseudomonas aeruginosa*, one strain of *Enterobacter hormaechei* and one strain of *Pseudomonas fluorescens* were used for further studies.

**Reviewer comment: **Were the three strains of *Pseudomonas aeruginosa* and the one strain of *Enterobacter hormaechei* further tested for their likely hood to be cross-kingdom pathogens? This has to be confirmed by whole genome sequencing and other phylogenetic studies because these bacteria in the current study cannot be used for agriculture application as suggested by the authors. These should not have even gone into the plant experiments before confirming their pathogenic status.

 “3.3.2 Effect of stepping duration” should be steeping duration.

 Instead of using so many crops, the authors could have focussed on 2 crops with proper experimental design to suggest how the bacterial cell-free supernatant components caused the growth effect.

 The results section of the germination experiment is not written well.

 The figures representing the culture age and IAA production in the absence and presence of tryptophan must be grouped in to one figure.

**In the manuscript: 2.4 Characterization of isolates and 2.7 Extraction and characterization of metabolites: **These sections in the materials and methods are not followed up in the results section.

 This manuscript is incomplete.

Is the work clearly and accurately presented and does it cite the current literature?

No

If applicable, is the statistical analysis and its interpretation appropriate?

Partly

Are all the source data underlying the results available to ensure full reproducibility?

No

Is the study design appropriate and is the work technically sound?

No

Are the conclusions drawn adequately supported by the results?

No

Are sufficient details of methods and analysis provided to allow replication by others?

No

Reviewer Expertise:

Molecular Plant-microbe interaction, Multi-omics of plants and microbes

I confirm that I have read this submission and believe that I have an appropriate level of expertise to state that I do not consider it to be of an acceptable scientific standard, for reasons outlined above.

# Response

**Published:** 2026-06-22

**Authors:** Damilola Abiodun

**Affiliations:** Biological Sciences, Afe Babalola University, Ado Ekiti, Ekiti State, Nigeria

**Point-Point Response to Reviewer**

**Reviewer Comment**

 The study focuses on the use of phytotoxic rhizobacteria to control weeds. This is a sustainable way of keeping the weeds under check. This study however has flaws in experimental design and the manuscript is not complete.

**Possible Response**

 We thank the reviewer for the valuable observations. The manuscript has been extensively revised to improve methodological clarity, correct reporting inconsistencies, and provide additional explanations regarding experimental design and interpretation of results. Missing sections have been expanded and results corresponding to all methods described have now been incorporated or appropriately removed where data were unavailable.

**Reviewer Comment**

 Some of the bacteria mentioned in the introduction, especially *Pseudomonas aeruginosa*, *Pseudomonas syringae* and *Enterobacter hormaechei* are also cross kingdom infectors. Without proper validation, these should not be even tested for a study such as this.

**Response**

 We appreciate this important biosafety concern. We acknowledge that certain species identified in this study have been reported as opportunistic pathogens or cross-kingdom infectors. The objective of this study was exploratory screening for phytotoxic potential and not development of field-ready bioherbicides. Accordingly, the manuscript has been revised to clearly state that the isolates are preliminary candidates requiring comprehensive biosafety assessment, including whole genome sequencing, virulence gene screening, pathogenicity testing and phylogenomic analyses before any agricultural application can be considered.

**Reviewer Comment**

 Please load a manuscript with lines numbered so that it is easier to review.

**Response**

 Thank you. The revised manuscript has been reformatted to include continuous line numbering throughout the document.

**Reviewer Comment**

 Check the SI units for correct representation. Eg., ml must be mL.

**Response**

 All units have been revised according to SI conventions.

**Reviewer Comment**

 Why was a universal approach taken to surface sterilize seeds? Five minutes in 5% sodium hypochlorite may affect cucumber and soybean embryos.

**Response**

 We thank the reviewer for this observation. The sterilization protocol was adopted from previous studies and was intended to ensure complete removal of surface contaminants across all seed types. However, we acknowledge that seed sensitivity may vary among species. To address this limitation, we have added a statement acknowledging the possibility of differential responses of seed types to the sterilization treatment and its potential influence on germination outcomes.

**Reviewer Comment**

 Were the germination experiments done only with 7 seeds per treatment?. This is difficult to defend scientifically.

**Response**

 We appreciate this observation. Seven seeds per replicate were used following the screening protocol described by Akpor et al. (2022), and the experiment was conducted in triplicate. However, we acknowledge that larger sample sizes are generally recommended for germination studies and would improve statistical robustness. The present study should therefore be considered a preliminary screening investigation rather than a definitive germination assessment.

**Reviewer Comment**

 What was the logic behind soaking 200 seeds but only 7 seeds taken for the germination experiment?

**Response**

 The 200 seeds were used exclusively for viability assessment and elimination of non-viable seeds prior to experimentation. Germination assays were subsequently conducted using representative viable seeds according to the screening protocol.

**Reviewer Comment**

 Two 100:0 dilutions were listed.

**Response**

 We thank the reviewer for identifying this typographical error. We have revised made the correction

**Reviewer Comment**

 Were the three strains of P. aeruginosa and E. hormaechei tested for cross-kingdom pathogenicity?

**Response**

 No. Whole-genome sequencing and virulence analyses were beyond the scope of the current study. This limitation has now been acknowledged explicitly in the revised manuscript.

**Reviewer Comment**

 "Stepping duration" should be "Steeping duration".

**Response**

 Corrected throughout the manuscript.

**Comment on Too Many Crops**

**Reviewer Comment**

 Authors should have focused on fewer crops.

**Response**

 We appreciate this suggestion. Multiple crops were included to provide a broad preliminary assessment of phytotoxic activity across seeds differing in physiological characteristics. Nevertheless, future studies will focus on fewer crops and investigate underlying phytotoxic mechanisms in greater detail.

**Reviewer Comment**

 The results section of the germination experiment is not written well.

**Response**

 The germination results section has been rewritten to improve clarity, reduce repetition and emphasize statistically significant findings rather than describing every numerical observation.

**IAA Figures**

**Reviewer Comment**

 Figures representing IAA production with and without tryptophan should be grouped.

**Response**

 We thank the reviewer for this valuable suggestion. We carefully considered combining the figures into a single multi-panel presentation. However, the figures were intentionally retained as separate figures to enhance clarity and facilitate independent interpretation of IAA production under the two experimental conditions. Since tryptophan supplementation substantially influenced the magnitude and pattern of IAA production among the isolates, presenting the datasets separately allows readers to more clearly visualize treatment-specific responses without overcrowding the figure or compromising readability. We believe that maintaining separate figures improves data presentation and minimizes potential ambiguity in comparing the effects of culture age under tryptophan-supplemented and non-supplemented conditions.

**Reviewer Comment**

 Sections 2.4 and 2.7 are not followed up in Results.

**This is a major issue.**

**Response**

 We thank the reviewer for identifying this omission. Gel image has been added to confirm section 2.4 while 2.7 has been removed.

**Reviewer Comment**

 Conclusions are not adequately supported by results.

**Response**

 The conclusion has been revised to avoid overstatement.
